# Emergence of *Stilbocrea gracilipes* associated with canker and dieback in pomegranate and eucalyptus trees and host-specific responses

**DOI:** 10.1128/spectrum.02839-25

**Published:** 2026-02-03

**Authors:** Pouria Sekandarpour, Hamed Negahban, Reza Mostowfizadeh-Ghalamfarsa

**Affiliations:** 1 Department of Plant Protection, School of Agriculture, Shiraz University, Shiraz, Iran; USDA-ARS San Joaquin Valley Agricultural Sciences Center, Parlier, California, USA

**Keywords:** *Bionectriaceae*, canker, cross-pathogenicity, cultivar susceptibility, dieback, ornamental trees, principal component analysis (PCA)

## Abstract

The increasing incidence of canker and dieback symptoms in commercially important fruit orchards and adjacent ornamental trees in Fars Province, Iran, prompted an investigation into associated fungal pathogens. Surveys in 2023 and 2024 yielded several isolates from symptomatic pomegranate (*Punica granatum* L.) and eucalyptus (*Eucalyptus camaldulensis* Dehnh.) trees, which were identified as *Stilbocrea gracilipes* based on morphological, physiological, and multi-locus phylogenetic analyses (ITS, *tef1-α*, and *rpb2* loci). Pathogenicity tests showed that all recovered *S. gracilipes* isolates (*n* = 23) cause dark brown lesions and wood discoloration in detached shoots of their respective hosts, with significant variation in aggressiveness. Cross-pathogenicity assessments using the most aggressive isolates on pomegranate and eucalyptus saplings revealed that isolates from original hosts were pathogenic to both plant species, inducing characteristic lesions and internal wood discoloration. Based on an assessment of eight pathogenicity traits, the pomegranate-obtained isolate (PM7-68) demonstrated more aggressiveness compared to the eucalyptus-obtained isolate (EU1-031). Furthermore, inoculated pomegranate saplings exhibited greater susceptibility than inoculated eucalyptus saplings, highlighting the potential threat of *S. gracilipes* to economically significant pomegranate orchards. Evaluation of pomegranate cultivar susceptibility to *S. gracilipes* isolates, using principal component analysis (PCA) and heatmap visualization, revealed clustering of disease severity into four distinct groups. The pomegranate cultivar ‘Malas-e-Danesiyah-e-Esfahani’ consistently exhibited high disease severity (level 4), identifying it as highly susceptible, while the cultivar ‘Kadro’ displayed reduced lesion development and slower disease progression (level 1), suggesting relative resistance.

**IMPORTANCE:**

This study represents the first report of *Stilbocrea gracilipes* associated with canker and dieback on pomegranate and eucalyptus, and the first confirmation of its pathogenicity as a canker-causing fungus on woody hosts in Iran and globally. The ability of *S. gracilipes* to infect both fruit and ornamental trees, coupled with its cross-host infectivity, poses a potential risk to mixed cropping systems and natural ecosystems.

## INTRODUCTION

Pomegranate (*Punica granatum* L.) and *Eucalyptus* spp., belonging to the *Lythraceae* and *Myrtaceae* families, respectively, exhibit broad adaptability, thriving in diverse agro-climatic zones ranging from tropical to subtropical regions. Pomegranate, originating from Iran and neighboring regions, displays significant genetic diversity (1) and remarkable resilience to arid conditions, requiring minimal water and tolerating poor soils (1, 2). In 2022, Iran produced about 1.1 million tons of pomegranates in approximately 96,000 ha, accounting for nearly one-fifth of global production (3, 4). As a valuable export crop with increasing international demand (5), pomegranate offers economic benefits from fruit consumption and processing to medicinal and industrial applications of other plant parts (6, 7). Similarly, eucalyptus, native to Australia and adjacent Pacific islands, has been widely introduced globally (8). Its rapid growth, drought tolerance, and desirable wood properties have made it a popular choice for forest plantations (9), serving as shade trees, ornamentals, and windbreaks (10). Over the last three decades, eucalyptus plantations have expanded worldwide, covering approximately 22.57 million hectares (11). Beyond timber, eucalyptus trees provide valuable non-timber products, such as essential oils and honey, with industrial applications in paper, furniture, energy, charcoal, and housing (12, 13). However, the initial success of eucalyptus trees outside their native range, attributed to a temporary escape from natural enemies, has been challenged by the emergence of diseases and pests, threatening the long-term sustainability of eucalyptus plantations (8, 14, 15).

In pomegranate, several fungal taxa have been identified in association with canker and dieback symptoms, including members of the *Bionectriaceae* (16), *Botryosphaeriaceae* (1, 2, 16–21), *Cytosporaceae* (22), *Diatrypaceae* (16, 23, 24), *Didymellaceae*, *Didymosphaeriaceae* (16), and *Togniniaceae* (16, 25, 26), with reports originating from various geographical locations including Australia, China, Greece, Iran, South Africa, and the USA. Similarly, eucalyptus canker diseases are attributed to multiple fungal pathogens, notably within the *Botryosphaeriaceae* (13, 27, 28), *Cryphonectriaceae* (9, 29–32), and *Teratosphaeriaceae* (8).

Among the diverse fungal taxa associated with canker and dieback diseases in woody hosts, members of the genus *Stilbocrea* (*Bionectriaceae*) have garnered increasing attention due to their pathogenic roles across a wide range of plant species (33–36). The diseases induced by *Stilbocrea* spp. manifest in various forms, such as wood discoloration and necrosis, canker development, and dieback (34–36). For instance, *S. banihashemiana* Bolboli, Tavakolian & Mostowf. is documented as a causal agent of canker and dieback in several fruit and ornamental species, including *Berberis vulgaris* L. (33), *Eriobotrya japonica* (Thunb.) Lindl., *Ficus carica* L. (34), *Juglans regia* L. (35), and *Rosa hybrida* L. (36). Similarly, *S. colubrensis* Lechat & J. Fourn. has been reported in association with *Bambusa vulgaris* Schrad. ex J.C.Wendl. (37). In addition, *S. gracilipes* (Tul. & C. Tul.) Samuels & Seifert has been isolated from a range of substrates, including twigs, bark, and dead wood of both dicotyledonous plants and palms, and dieback symptoms in chico (*Manilkara zapota* [L.] P.Royen), black locust (*Robinia pseudoacacia* L.), and saxaul (*Haloxylon* spp.) (38–42). *Stilbocrea macrostoma* (Berk. & M.A. Curtis) Höhn. has been found in connection with wood necrosis affecting *Quercus brantii* Lindl. (43). Furthermore, *S. walteri* Voglmayr & Jaklitsch has been identified in association with *Quercus ilex* L. (44) and has also been linked to canker and dieback diseases in *Citrus* spp. (45) and *P. granatum* (16) trees.

During recent surveys of canker and dieback diseases in pomegranate orchards of Fars Province, one of Iran’s major pomegranate-producing regions, and on landscape ornamental eucalyptus (*Eucalyptus camaldulensis* Dehnh.) trees, several *Bionectriaceae*-like isolates were recovered from cankers, dieback, and discolored internal wood of both pomegranate and eucalyptus trees. Based on morphological characteristics, these isolates were tentatively identified as members of the *Stilbocrea* genus. The current study aimed to: (i) characterize the morphological and cultural features of these *Bionectriaceae*-like isolates, as well as multi-locus phylogenetic analyses; (ii) assess the pathogenicity and aggressiveness of all obtained isolates on detached shoots of their respective hosts; (iii) evaluate the cross-pathogenicity of isolates on the natural hosts; and (iv) assess the susceptibility of eight pomegranate cultivars to these isolates through various pathogenicity traits and robust statistical analyses.

## RESULTS

### Field surveys, disease symptoms, and fungal isolates

**Fig 1:**
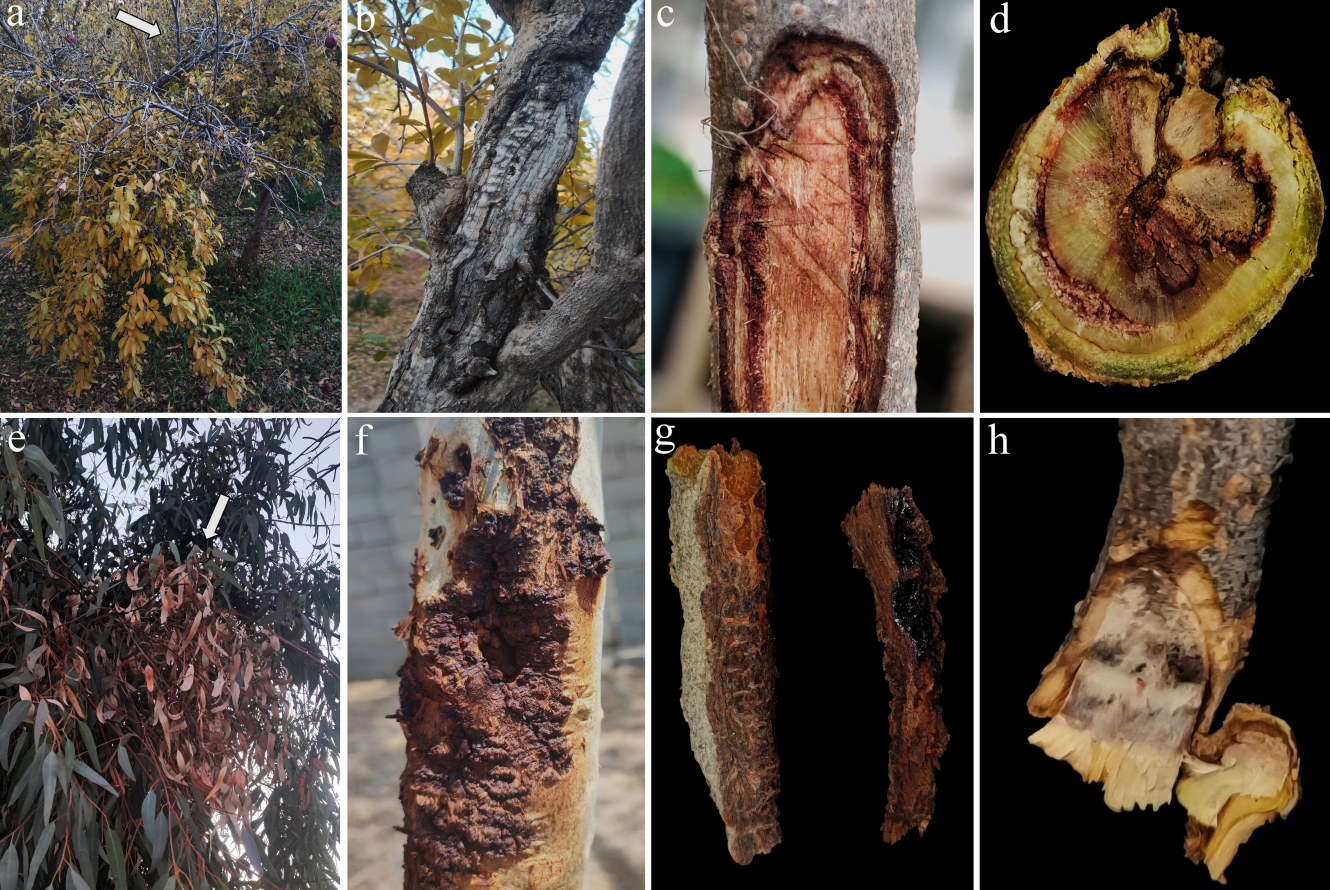
Symptoms observed in association with *Stilbocrea gracilipes* infection on pomegranate and eucalyptus trees in Fars Province, Iran. (**a–d**) Symptomatic pomegranate samples. (**e–h**) Symptomatic eucalyptus samples. (**a and e**) Dieback and overall decline. (**b, c, f, and g**) Canker formation and wood necrosis. (**d and h**) Internal wood discoloration of one-year-old branches.

Field surveys of commercial pomegranate orchards and eucalyptus trees in Fars Province, southern Iran, revealed a widespread syndrome characterized by branch and trunk cankers, twig dieback, internal discoloration, and overall decline. Our observations indicated a higher incidence of this syndrome in older, established plantations compared to newly established, well-maintained orchards and landscaped areas. Pomegranate trees exhibited external symptoms, including sunken, elongated necrotic lesions on trunks and mature branches, often with bark cracking and callus formation (Fig. 1). Severe infections resulted in progressive dieback, yellowing, premature leaf drop and necrosis, general wood tissue decay, and eventual tree death. Eucalyptus trees displayed necrotic cankers with bark splitting and gummosis on the main trunk and branches. Internal symptoms in both species included wood discoloration, sectorial vascular necrosis, and dark brown spots in vascular tissue observed in cross-sections of symptomatic trunk and branches (Fig. 1). From a collection of 50 ascomycete isolates obtained from symptomatic pomegranate (33 isolates) and eucalyptus (17 isolates) trees, 23 isolates were grouped in a *Bionectriaceae*-like morphotype, producing terminal and lateral phialides and abundant allantoid and ovoid conidia (Table 1) (46). This subset represents 45.4% of the isolates from pomegranate and 47.1% of the isolates from eucalyptus. Other recovered isolates exhibited morphological characteristics consistent with members of the *Didymellaceae*, *Nectriaceae*, and *Pleosporaceae* families. The *Bionectriaceae*-like isolates originated from 10 infected pomegranate trees in two orchards located in Shiraz (12 isolates), an orchard in Arsanjan County (three isolates), and from four eucalyptus trees at two separate sites in Shiraz County (eight isolates). From pomegranate, 60% of isolates were recovered from symptomatic mature branches, 26.7% from one-year-old branches, and 13.3% from the trunk. In eucalyptus, 50% of isolates originated from mature branches, 25% from one-year-old branches, and 25% from the trunk.

**TABLE 1:** Information of *Stilbocrea gracilipes* isolates collected from infected pomegranate and eucalyptus trees in Fars Province, Iran^*a*^

Isolate code	Isolate source	Date	Disease symptom(s)	Plant species	Location	Latitude	Longitude
PM2-51	Mature branch	2025-01-04	C, D, IWD	*Punica granatum* cv. ‘Rabab-e-Neyriz’	Shiraz	29.68071	52.4716
PM3-25	Mature branch	2025-01-04	C, D	*P. granatum* cv. ‘Rabab-e-Neyriz’	Shiraz	29.68061	52.47161
PM5-62	Trunk	2025-01-04	C, D, IWD	*P. granatum* cv. ‘Rabab-e-Neyriz’	Shiraz	29.68054	52.47166
PM6-63	One-year-old branch	2025-01-04	C, IWD	*P. granatum* cv. ‘Rabab-e-Neyriz’	Shiraz	29.68028	52.47189
PM7-68	Mature branch	2025-01-04	C, D, IWD	*P. granatum* cv. ‘Rabab-e-Neyriz’	Shiraz	29.68015	52.47198
PM7-61	Mature branch	2025-01-04	C, IWD, WD	*P. granatum* cv. ‘Rabab-e-Neyriz’	Shiraz	29.68015	52.47198
PM7-35	Trunk	2025-01-04	C, D, IWD	*P. granatum* cv. ‘Rabab-e-Neyriz’	Shiraz	29.68015	52.47198
PM8-41	Mature branch	2025-01-04	C, IWD	*P. granatum* cv. ‘Rabab-e-Neyriz’	Shiraz	29.68006	52.47206
PM8-33	One-year-old branch	2025-01-04	C, D, IWD	*P. granatum* cv. ‘Rabab-e-Neyriz’	Shiraz	29.68006	52.47206
PM9-62	Mature branch	2025-01-04	C, TD, EWD	*P. granatum* cv. ‘Rabab-e-Neyriz’	Shiraz	29.68056	52.4733
PM9-45	Mature branch	2025-01-04	CTD, EWD	*P. granatum* cv. ‘Rabab-e-Neyriz’	Shiraz	29.68056	52.4733
PM9-36	Mature branch	2025-01-04	C, D, IWD, EWD	*P. granatum* cv. ‘Shirin-e-Shahvar’	Shiraz	29.68056	52.4733
APM1-01	One-year-old branch	2025-01-25	C, IWD	*P. granatum* cv. ‘Rabab-e-Neyriz’	Arsanjan	29.88521	53.39307
APM2-01	One-year-old branch	2025-01-25	IWD	*P. granatum* cv. ‘Rabab-e-Neyriz’	Arsanjan	29.88519	53.39305
APM3-06	Mature branch	2025-01-25	C, D, IWD	*P. granatum* cv. ‘Rabab-e-Neyriz’	Arsanjan	29.88511	53.39291
EU1-031	Trunk	2024-02-18	C, D, IWD, TF	*Eucalyptus camaldulensis*	Shiraz	29.55842	52.59493
EU1-051	Trunk	2024-02-18	IWD, TD, TF	*E. camaldulensis*	Shiraz	29.55842	52.59493
EU1-01	Mature branch	2024-02-18	IWD, TD, EWD	*E. camaldulensis*	Shiraz	29.55842	52.59493
EU1-014	Mature branch	2024-02-18	C, IWD, EWD	*E. camaldulensis*	Shiraz	29.55842	52.59493
EU2-071	One-year-old branch	2024-02-18	C, IWD	*E. camaldulensis*	Shiraz	29.55876	52.59498
EU2-081	Mature branch	2024-02-18	C, D, IWD	*E. camaldulensis*	Shiraz	29.55876	52.59498
EU3-04	One-year-old branch	2024-02-18	C, D, IWD	*E. camaldulensis*	Shiraz	29.68019	52.47202
EU4-02	Mature branch	2024-02-18	C, TD, EWD	*E. camaldulensis*	Shiraz	29.68052	52.47168

^*a*^ C, canker; D, decline; IWD, internal wood discoloration; TD, twig dieback; TF, trunk flaking; EWD, external wood discoloration.

### Morphological characterizations and the effect of temperature on mycelial growth

**Fig 2:**
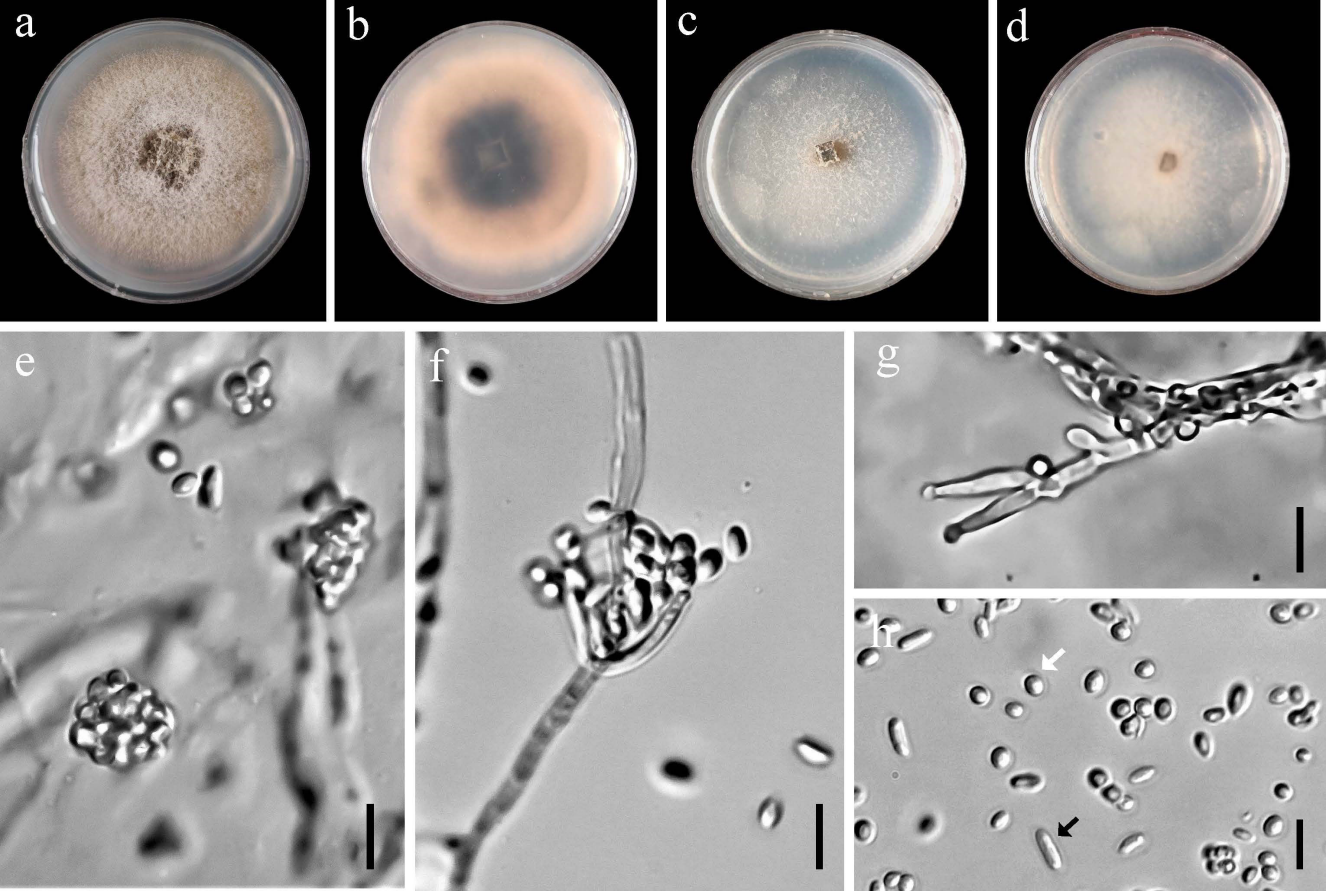
Cultural and asexual structures of *Stilbocrea gracilipes* obtained from symptomatic pomegranate and eucalyptus trees in Fars Province, Iran. (**a and b**) Fungal colony grown on PDA for 14 days at 25°C under a 12-hour light/dark cycle, surface view, and reverse view, respectively. (**c and d**) Colony on MEA incubated under identical conditions, surface view, and reverse view, respectively. (**e**) Dense, ovoid conidia on phialides. (**f and g**) Lageniform to cylindrical phialides, both terminal and lateral, borne on branched conidiophores. (**h**) Unicellular, smooth, hyaline conidia appearing in two distinct shapes: ovoid (white arrow) and allantoid (black arrow). Scale bar = 5 μm.

**Fig 3:**
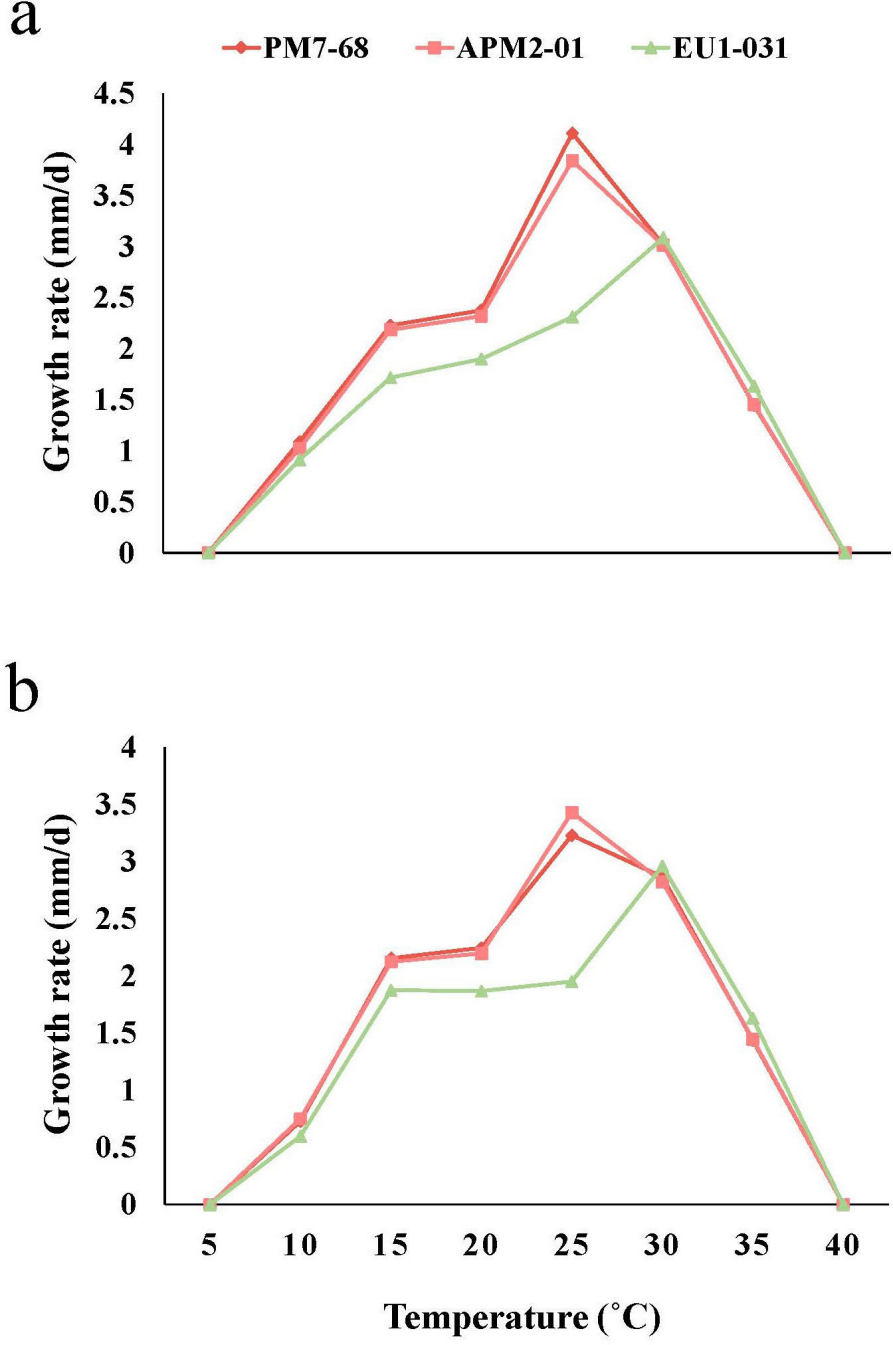
Average mycelial growth rate of *Stilbocrea gracilipes* isolates, recovered from infected pomegranate (PM7-68 and APM2-01) and eucalyptus (EU1-031) trees, measured at eight different temperatures. (**a**) Isolates on potato dextrose agar (PDA) and (**b**) on malt extract agar (MEA).

On potato dextrose agar (PDA), *Bionectriaceae*-like isolates initially produced cottony, white aerial mycelia, which subsequently developed olivaceous-gray centers, olivaceous-buff middle rings, and white margins. A similar pattern was observed on MEA, with colonies starting as creamy-white before developing dense white aerial mycelium centrally and scattered peripheral mycelial formations. Over time, the aerial mycelium transitioned to an olive-green coloration (Fig. 2). Conidia were abundant, hyaline, and smooth-walled, ovoid to allantoid, and were either straight or slightly curved (Fig. 2). Allantoid conidia measured 3.73–6.61 × 1.46–2.76 µm (average 4.73 ± 0.6 × 1.96 ± 0.24 µm; *n* = 90), while ovoid conidia ranged from 1.96 to 2.99 × 1.88–2.62 µm (average 2.37 ± 0.26 × 2.28 ± 0.22 µm; *n* = 90). Phialides were terminal and lateral, abundant on aerial mycelium, branched, wide at the base, and ranged from cylindrical to lageniform in shape (Fig. 2), measuring 6.3–9.43 × 1.94–3.18 µm (average 8.57 ± 0.24 × 2.48 ± 0.2 µm; *n* = 90). Conidiophores branched once or twice monochasially. Temperature-growth relationship assays revealed minimum growth rates at 10°C and an optimal growth rate at 25°C (for APM2-01 and PM7-68) and 30°C (for EU1-031) on both PDA and MEA (Fig. 3). Moreover, no growth was observed on PDA and MEA at 5 and 40°C. At 25°C on PDA, radial growth rates ranged from 2.31 to 4.11 mm/day (average 3.42 ± 0.5 mm/day), with colonies reaching an average diameter of 47.88 mm after 14 days under a 12-hour photoperiod. On MEA at 25°C, radial growth rates were recorded at 1.95 to 3.43 mm/day (average 2.87 ± 0.3 mm/day), with colonies reaching an average diameter of 40.18 mm within 14 days. These morphological characteristics were consistent with members of *Stilbocrea* spp. (37–39).

### Molecular identification and phylogenetic analyses

**Fig 4:**
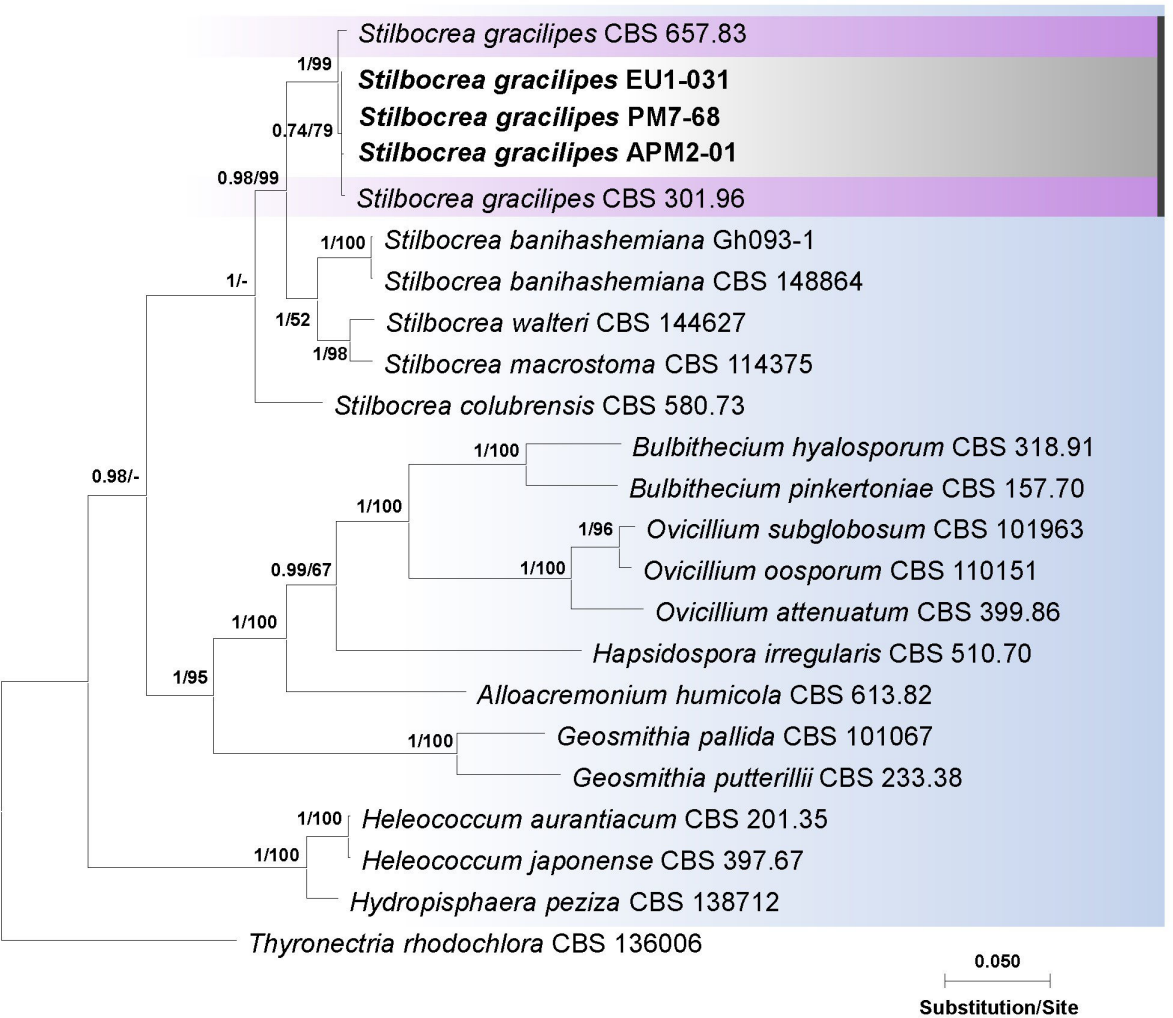
Phylogenetic relationships of *Stilbocrea gracilipes* isolates obtained from infected pomegranate and eucalyptus trees in southern Iran. The Bayesian tree, based on a combined data set of internal transcribed spacers 1 and 2, the 5.8S gene of rDNA (ITS), translation elongation factor 1-α (*tef1-α*), and the second largest subunit of RNA polymerase II (*rpb2*), illustrates the phylogenetic placement of isolates APM2-01, EU1-031, and PM7-68 within the *Bionectriaceae* family. Bootstrap support values from the maximum likelihood analysis (ML-BS, right) and Bayesian posterior probabilities (BI-PP, left) are shown at the nodes. Branches with ML-BS = 100 and BI-PP = 1 are considered fully supported. The tree was rooted using *Thyronectria rhodochlora* (CBS 136006). Isolates from this study are shown in bold.

Sequence comparisons using BLASTn revealed a high degree of nucleotide identity (exceeding 99%) between the ITS, *tef1-α*, and *rpb2* sequences of representative isolates (APM2-01, EU1-031, and PM7-68; Table 1) obtained from diseased pomegranate and eucalyptus trees and *S. gracilipes* isolates (CBS 301.96 and CBS 657.83; Table S2) (46). Phylogenetic analyses employing both Bayesian inference (BI) and maximum likelihood (ML) methods, based on individual loci (ITS, *tef1*, and *rpb2*) and their concatenated alignment, demonstrated that the *Stilbocrea* sp. isolates from this study clustered within a well-supported monophyletic lineage within the *Stilbocrea* clade of *Bionectriaceae*, closely related to *S. gracilipes* isolates (Fig. 4; Fig. S1 to S3). In the concatenated phylogenetic tree (Fig. 4), this lineage exhibited strong support, as evidenced by high bootstrap values (99% for ML) and posterior probabilities (1.00 for BI). The phylogenetic placement of the isolates obtained in this study, combined with their distinct morphological characteristics, provides robust support for their identification as *S. gracilipes*.

### Pathogenicity and aggressiveness assessments of isolates

**Fig 5:**
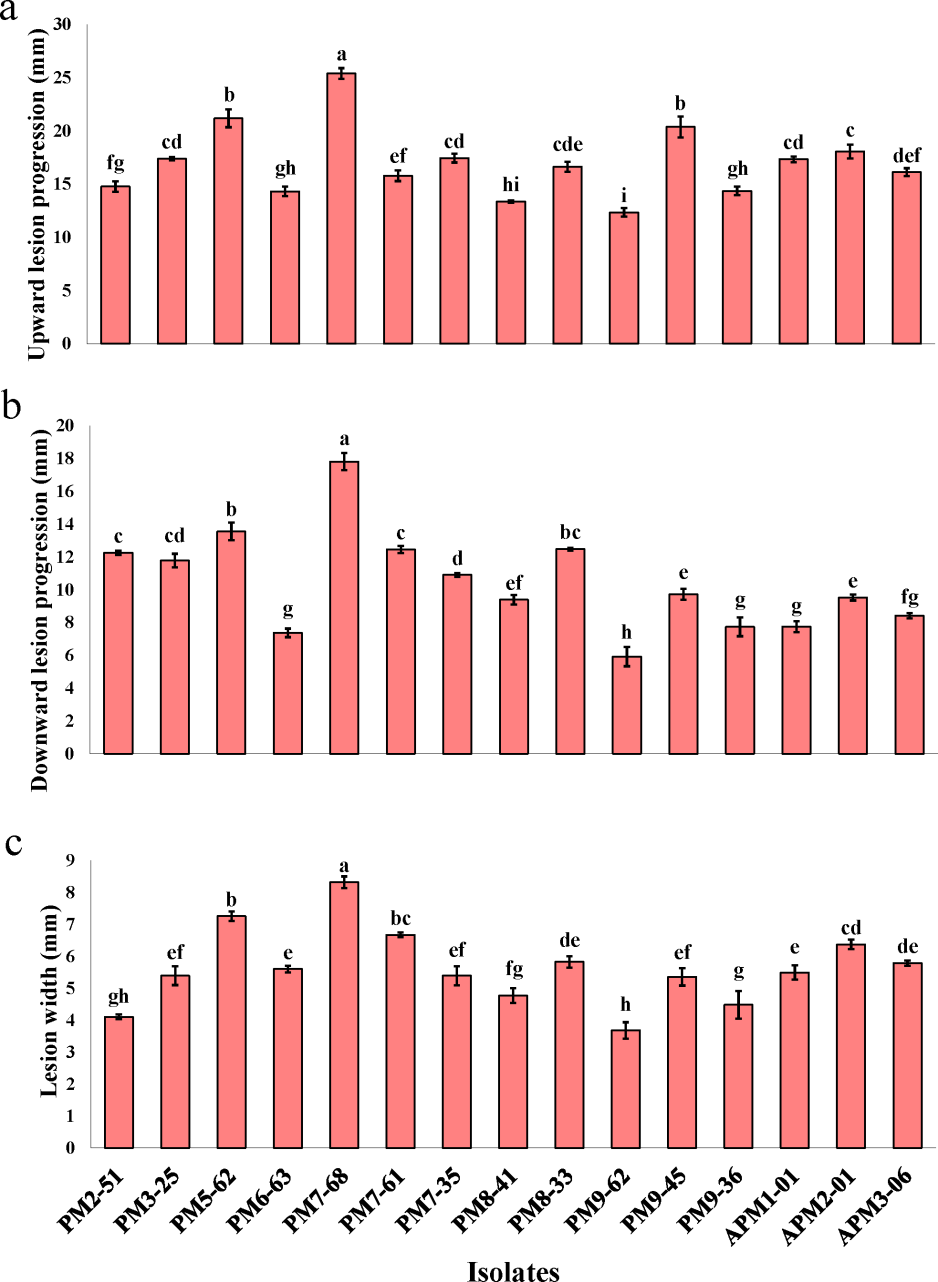
Pathogenicity traits of 15 *Stilbocrea gracilipes* isolates recovered from infected pomegranate trees and assessed via inoculation of detached pomegranate shoots. The figure illustrates the mean distribution of wood lesion progression: (**a**) upward direction, (**b**) downward direction, and (**c**) lesion width. Negative controls, inoculated with sterile PDA, remained asymptomatic. Statistically significant differences (*P* ≤ 0.05) are denoted by different letters adjacent to the values. Error bars represent standard deviations derived from three replicates.

**Fig 6:**
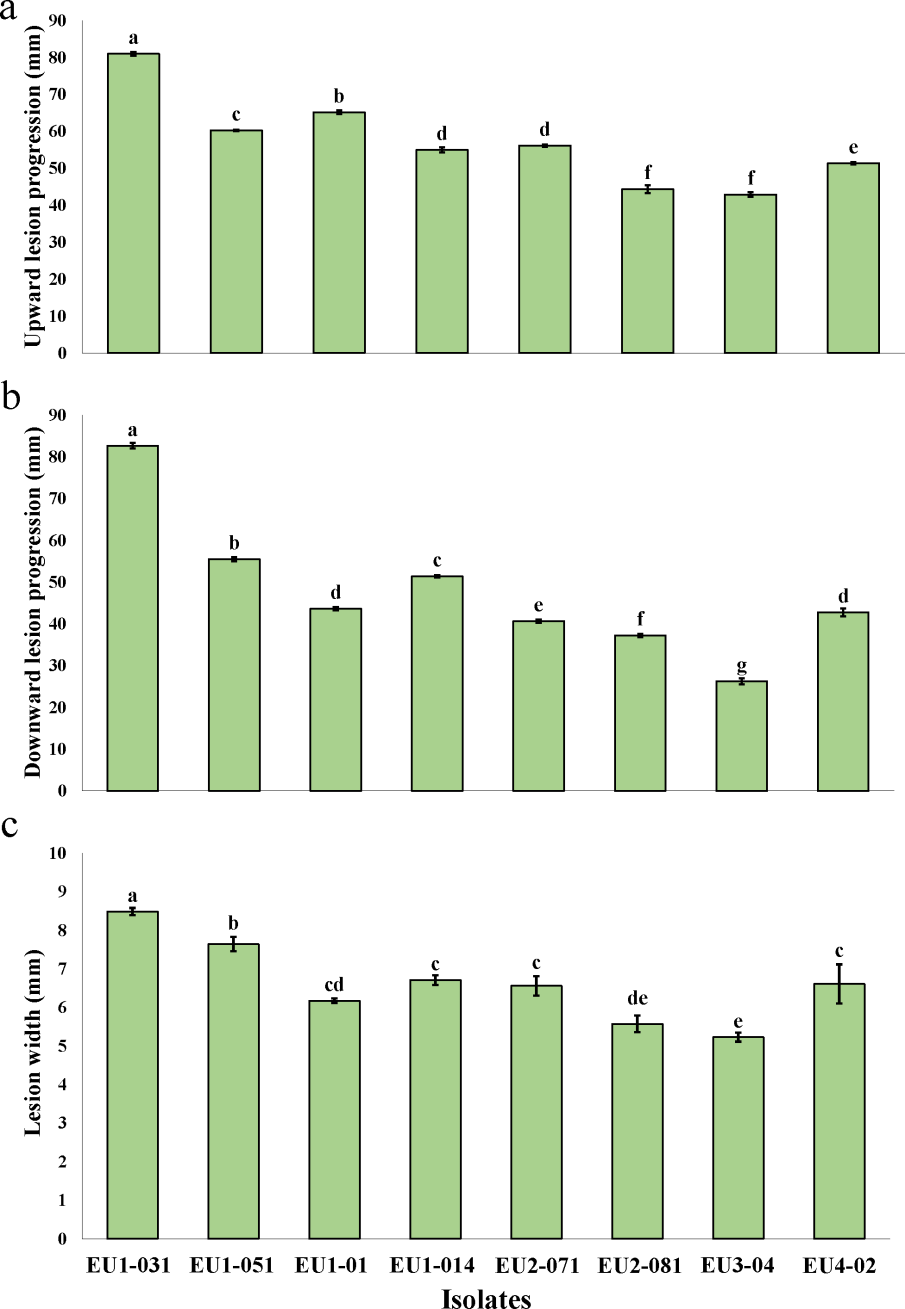
The pathogenicity of eight *Stilbocrea gracilipes* isolates obtained from infected eucalyptus trees was evaluated through inoculation of detached eucalyptus shoots. This figure presents the mean distribution of wood lesion progression: (**a**) upward direction, (**b**) downward direction, and (**c**) lesion width. Negative controls, inoculated with sterile PDA, remained asymptomatic. Statistically significant differences (*P* ≤ 0.05) among isolates are indicated by distinct letters adjacent to the corresponding mean values. Error bars represent the standard deviations calculated from three independent replicates.

In pathogenicity assays conducted on detached pomegranate and eucalyptus shoots, all 23 *S*. *gracilipes* isolates caused wood discoloration surrounding the inoculation sites by 15 days post-inoculation, confirming their pathogenic role on their respective hosts. Analysis of variance (ANOVA) demonstrated statistically significant differences (*P* < 0.0001) in pathogenicity traits (ULP: upward lesion progression; DLP: downward lesion progression; and LW: lesion width) among the isolates (Tables S3 and S4). Subsequent analysis using Tukey’s HSD test identified *S. gracilipes* isolate PM7-68 as the most aggressive on pomegranate shoots, resulting in the largest ULP (25.38 ± 0.5 mm), DLP (17.80 ± 0.53 mm), and LW (8.32 ± 0.2 mm). Conversely, isolate PM9-62 was the least aggressive, producing significantly smaller wood lesion size (Fig. 5). On eucalyptus shoots, isolate EU1-031 caused the most extensive necrotic wood lesions (ULP: 81.04 ± 0.46 mm, DLP: 82.67 ± 0.65 mm, LW: 8.48 ± 0.1 mm), while isolate EU3-04 resulted in the smallest wood lesion size (Fig. 6). Negative controls remained asymptomatic in inoculated shoots of both host species. The inoculated isolates were successfully re-isolated from the inoculated detached shoots, whereas no *S. gracilipes* isolates were recovered from the tissues of control shoots.

### Cross-pathogenicity evaluation of *S. gracilipes* isolates on one-year-old saplings of pomegranate and eucalyptus

**Fig 7:**
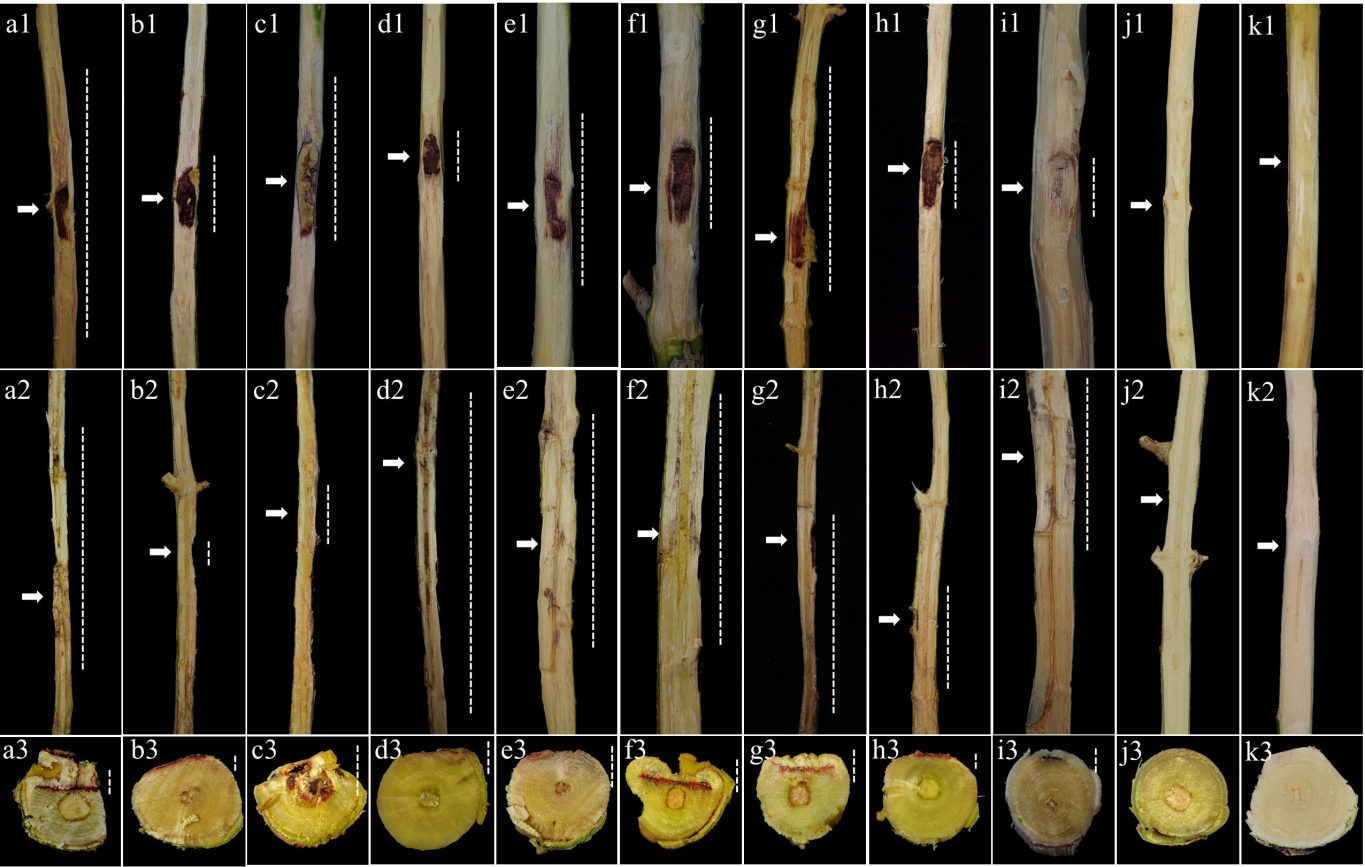
Pathogenicity assessment of *Stilbocrea gracilipes* isolate PM7-68 (recovered from a symptomatic pomegranate tree) on eight pomegranate cultivars and *Eucalyptus camaldulensis* saplings, two months after inoculation. (a1–k1) Necrosis and wood discoloration on inoculated one-year-old stems; (a2–k2) Longitudinal sections illustrating internal lesion development; and (a3–k3) cross-sections showing the extent of wood discoloration. Cultivars: (a1–a3) ‘Rabab-e-Neyriz’, (b1–b3) ‘Kadro’, (c1–c3) ‘Bihaste Ravar’, (d1–d3) ‘Malas-e-Danesiyah-e-Esfahani’, (e1–e3) ‘Malas-e-Saveh’, (f1–f3) ‘Shirin-e-Shahvar’, (g1–g3) ‘Wonderful’, (h1–h3) ‘Atabaki’, and (i1–i3) *E. camaldulensis*. Negative control saplings were inoculated with sterile PDA plugs on pomegranate (j1–j3) and eucalyptus (k1–k3). Arrows indicate inoculation points, and dotted lines represent lesion progression.

**Fig 8:**
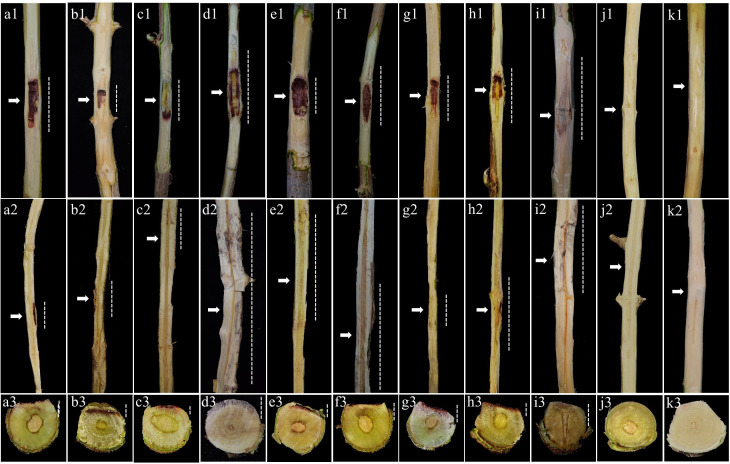
Pathogenicity assessment of *Stilbocrea gracilipes* isolate EU1-031 (recovered from a symptomatic eucalyptus tree) on eight pomegranate cultivars and *Eucalyptus camaldulensis* saplings, two months after inoculation. (a1– k1) Necrosis and vascular discoloration on inoculated stems; (a2k2) longitudinal sections illustrating internal lesion development; and (a3k3) cross-sections showing the extent of wood discoloration. Cultivars: (a1–a3) ‘Rabab-e-Neyriz’, (b1–b3) ‘Kadro’, (c1–c3) ‘Bihaste Ravar’, (d1–d3) ‘Malas-e-Danesiyah-e-Esfahani’, (e1–e3) ‘Malas-e-Saveh’, (f1–f3) ‘Shirin-e-Shahvar’, (g1–g3) ‘Wonderful’, (h1–h3) ‘Atabaki’, and (i1–i3) *E. camaldulensis*. Negative control saplings were inoculated with sterile PDA plugs on pomegranate (j1–j3) and eucalyptus (k1–k3) (repeated from Fig. 7). Arrows indicate inoculation points, and dotted lines represent lesion progression.

The cross-pathogenicity assays revealed that both *S. gracilipes* isolates, PM7-68 and EU1-031, exhibited pathogenicity on both pomegranate and eucalyptus one-year-old saplings. Irrespective of their origin, both isolates caused disease symptoms, demonstrating their capacity to infect both host species. Symptomatic saplings exhibited vascular discoloration and necrotic lesions, while control saplings remained asymptomatic (Fig. 7 and 8). Initial symptoms in inoculated saplings manifested as brown necrotic blotches at the inoculation site, which progressively expanded upward and downward from the inoculated sites. Disease progression was characterized by leaf yellowing, subsequent defoliation, and diminished growth within 30–45 days post-inoculation. These symptoms were consistently observed in both pomegranate and eucalyptus saplings, although variations in disease severity were noted. *Stilbocrea gracilipes* was successfully re-isolated from symptomatic tissues (isolation rate 88.9–100%), thus fulfilling Koch’s postulates and confirming its etiological role in stem canker and dieback development in both *P. granatum* and *E. camaldulensis*.

**Fig 9:**
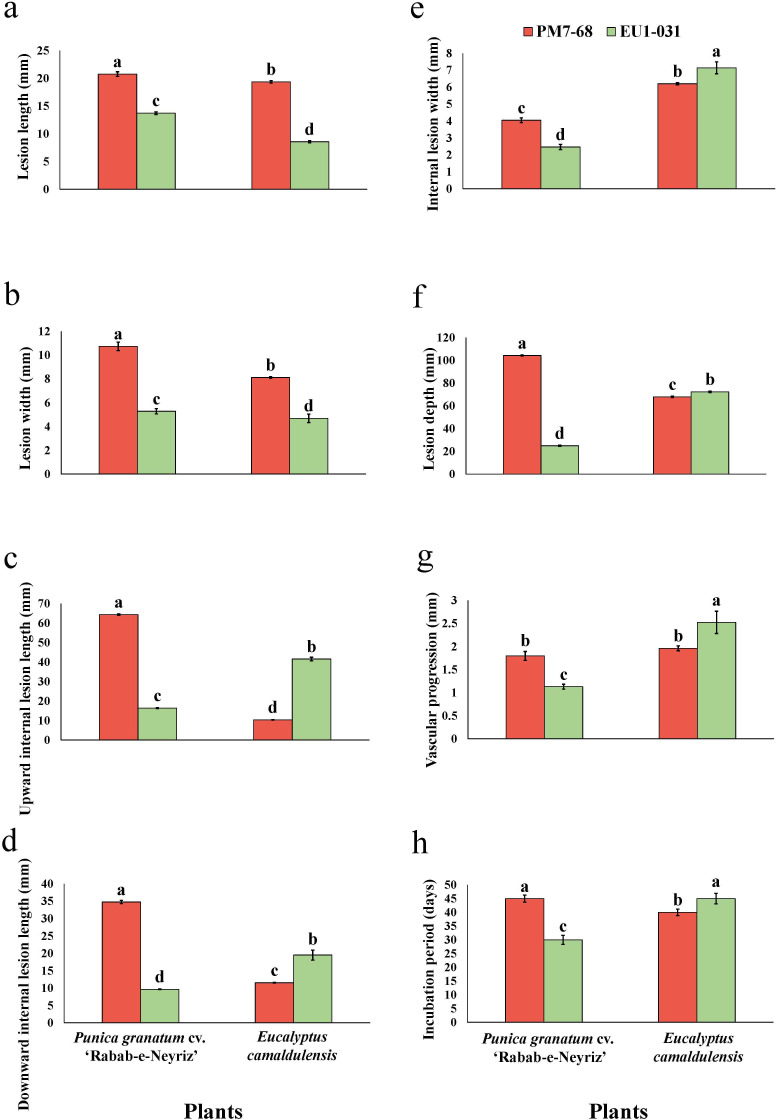
Assessment of lesion development on one-year-old saplings of *Eucalyptus camaldulensis* and the *Punica granatum* cv. ‘Rabab-e-Neyriz’ following inoculation with *Stilbocrea gracilipes* isolates derived from symptomatic tissues of pomegranate (PM7-68) and eucalyptus (EU1-031) trees. The graphs illustrate the mean values of: (**a**) lesion length; (**b**) lesion width; (**c**) upward internal lesion length; (**d**) downward internal lesion length; (**e**) internal lesion width; (**f**) lesion depth; (**g**) vascular progression; and (**h**) incubation period, measured 60 days post-inoculation. Statistically significant differences (*P* ≤ 0.05) among treatments are denoted by different letters. Error bars indicate standard deviations based on six biological replicates.

A factorial ANOVA revealed a significant influence of isolate, host species, and their interaction on all eight pathogenicity traits evaluated (*P* < 0.0001; Table S5). Subsequent analysis using Tukey’s HSD test demonstrated significant variation in pathogenicity traits across different host species-isolate combinations (Fig. 8). For example, the pomegranate-obtained isolate (PM7-68) exhibited the highest lesion length (LL) on pomegranate saplings (20.77 ± 0.43 mm), while the eucalyptus-obtained isolate (EU1-031) produced the shortest LL on eucalyptus saplings (8.57 ± 0.2 mm). Similarly, LW was greatest in pomegranate saplings inoculated with the PM7-68 isolate (10.74 ± 0.36 mm) and lowest in eucalyptus saplings inoculated with the EU1-031 isolate (4.69 ± 0.36 mm). Internal wood lesion development also varied significantly. The UILL reached its maximum in pomegranate saplings inoculated with the PM7-68 isolate (64.35 ± 0.4 mm), while the shortest UILL was observed in eucalyptus saplings inoculated with the same PM7-68 isolate (10.33 ± 0.12 mm). The downward internal lesion length (DILL) was greatest in pomegranate saplings inoculated with the PM7-68 isolate (34.76 ± 0.45 mm), and the lowest DILL was observed in pomegranate saplings inoculated with the EU1-031 isolate (9.64 ± 0.12 mm). In contrast, the internal lesion width (ILW) was largest in eucalyptus saplings inoculated with the EU1-031 isolate (7.14 ± 0.35 mm) but smallest in pomegranate saplings inoculated with the same isolate (2.47 ± 0.16 mm). Moreover, lesion depth (LD) was highest in eucalyptus saplings inoculated with the EU1-031 isolate (2.52 ± 0.24 mm) and lowest in pomegranate saplings inoculated with the EU1-031 isolate (1.13 ± 0.05 mm). The vascular progression (VP) was maximal in pomegranate saplings inoculated with the PM7-68 isolate (104.30 ± 0.48 mm) and minimal in pomegranate saplings inoculated with the EU1-031 isolate (24.94 ± 0.45 mm). The incubation period (IP) ranged from 30 to 45 days, with the shortest IP observed in pomegranate saplings inoculated with the EU1-031 isolate and the longest IP observed in eucalyptus saplings inoculated with the same EU1-031 isolate (Fig. 9).

### Susceptibility of pomegranate cultivars to *S. gracilipes* isolates

**Fig 10:**
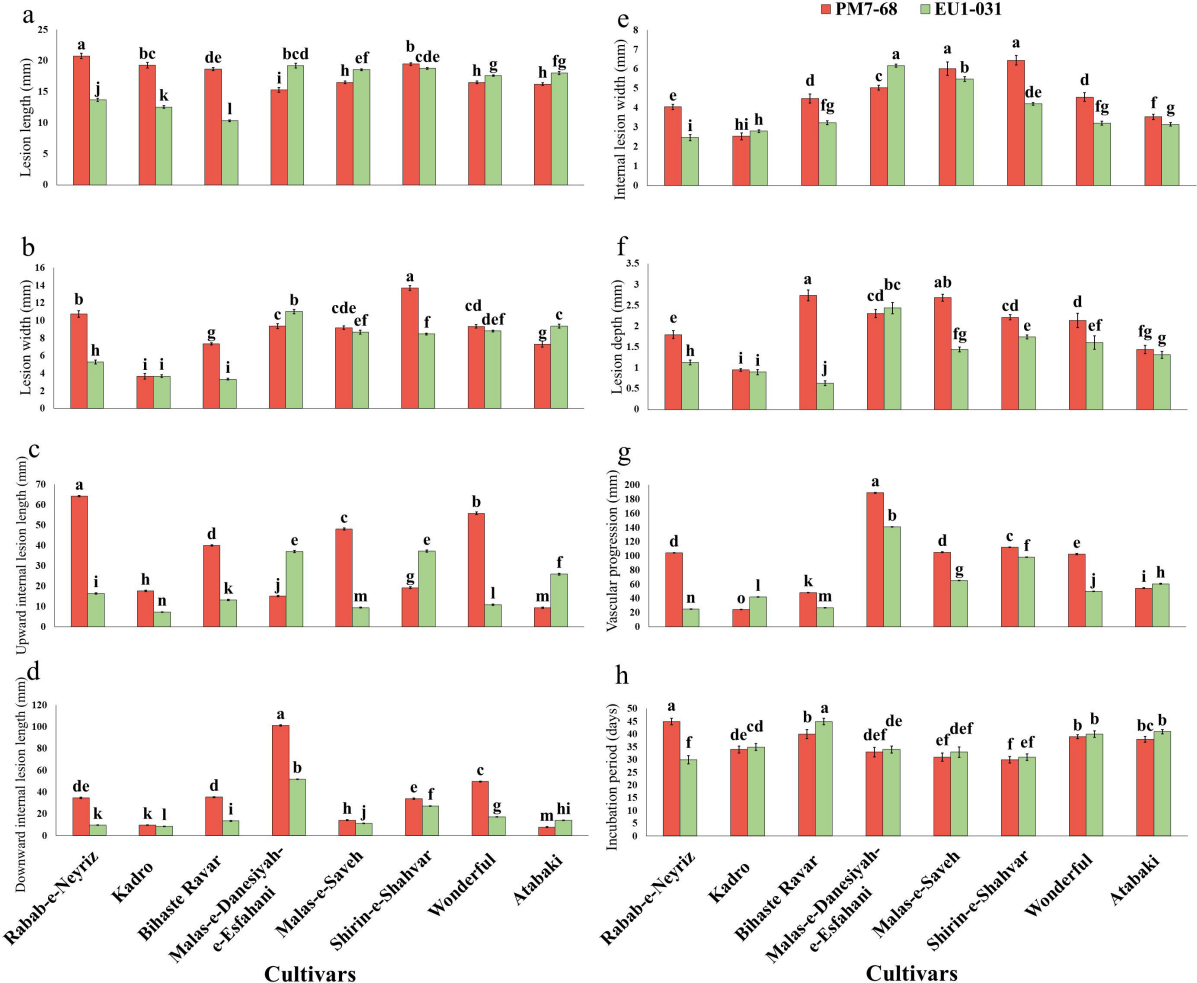
Pathogenicity traits evaluated on one-year-old saplings of eight commercial pomegranate cultivars, two months following artificial infection with *Stilbocrea gracilipes* isolates obtained from symptomatic pomegranate (PM7-68) and eucalyptus (EU1-031) wood tissues. The chart displays the mean: (**a**) lesion length; (**b**) lesion width; (**c**) upward internal lesion length; (**d**) downward internal lesion length; (**e**) internal lesion width; (**f**) lesion depth; (**g**) vascular progression; and (**h**) incubation period among cultivars. Different letters indicate statistically significant differences at *P* ≤ 0.05. Error bars represent standard deviations based on six biological replicates.

The susceptibility assessment of eight pomegranate cultivars to *S. gracilipes* revealed that isolates from both pomegranate and eucalyptus hosts induced disease symptoms on all cultivars within two months post-inoculation (Fig. 7 and 8). A factorial ANOVA revealed a significant interaction between isolate and cultivar on all eight evaluated pathogenicity traits (*P* < 0.0001; Table S6). Tukey’s HSD test further identified significant differences in these traits among the various cultivar-isolate interactions (Fig. 10). The pomegranate-obtained isolate (PM7-68) resulted in the largest LL in ‘Rabab-e-Neyriz’ saplings (20.77 ± 0.05 mm), while the eucalyptus-obtained isolate (EU1-031) produced the smallest LL in ‘Bihaste Ravar’ saplings (10.34 ± 0.13 mm). Similarly, the highest LW was observed in ‘Shirin-e-Shahvar’ saplings inoculated with PM7-68 (13.73 ± 0.04 mm), and the smallest LW in ‘Bihaste Ravar’ saplings inoculated with EU1-031 (3.33 ± 0.03 mm). For internal lesion parameters, the largest UILL was recorded in ‘Rabab-e-Neyriz’ saplings inoculated with PM7-68 (64.35 ± 0.03 mm), and the smallest UILL in ‘Kadro’ saplings inoculated with EU1-031 (7.28 ± 0.02 mm). The highest DILL was observed in ‘Malas-e-Danesiyah-e-Esfahani’ saplings inoculated with PM7-68 (101.34 ± 0.03 mm), while the smallest DILL was observed in ‘Kadro’ saplings inoculated with EU1-031 (8.47 ± 0.02 mm). The highest ILW was recorded in ‘Shirin-e-Shahvar’ saplings inoculated with PM7-68 isolate (6.45 ± 0.24 mm), while the smallest ILW was observed in ‘Rabab-e-Neyriz’ saplings inoculated with EU1-031 isolate (2.47 ± 0.05 mm). The PM7-68 isolate exhibited the greatest LD in ‘Bihaste Ravar’ saplings (2.74 ± 0.04 mm), whereas the smallest LD was recorded in the same cultivar inoculated with EU1-031 isolate (0.63 ± 0.04 mm). The VP varied significantly among treatments, with the highest VP observed in ‘Malas-e-Danesiyah-e-Esfahani’ saplings inoculated with the pomegranate isolate (189.15 ± 0.02 mm) and the lowest in ‘Kadro’ saplings inoculated with the same pomegranate isolate (24.28 ± 0.04 mm). The IP ranged from 30 to 45 days, with the longest IP observed in ‘Bihaste Ravar’ and ‘Rabab-e-Neyriz’ saplings inoculated with EU1-031 and PM7-68 isolates, respectively (45 ± 1.30 days). The shortest IP observed in ‘Shirin-e-Shahvar’ and ‘Rabab-e-Neyriz’ saplings inoculated with PM7-68 and EU1-031 isolates, respectively (30 ± 1.30 days) (Fig. 10).

**Fig 11:**
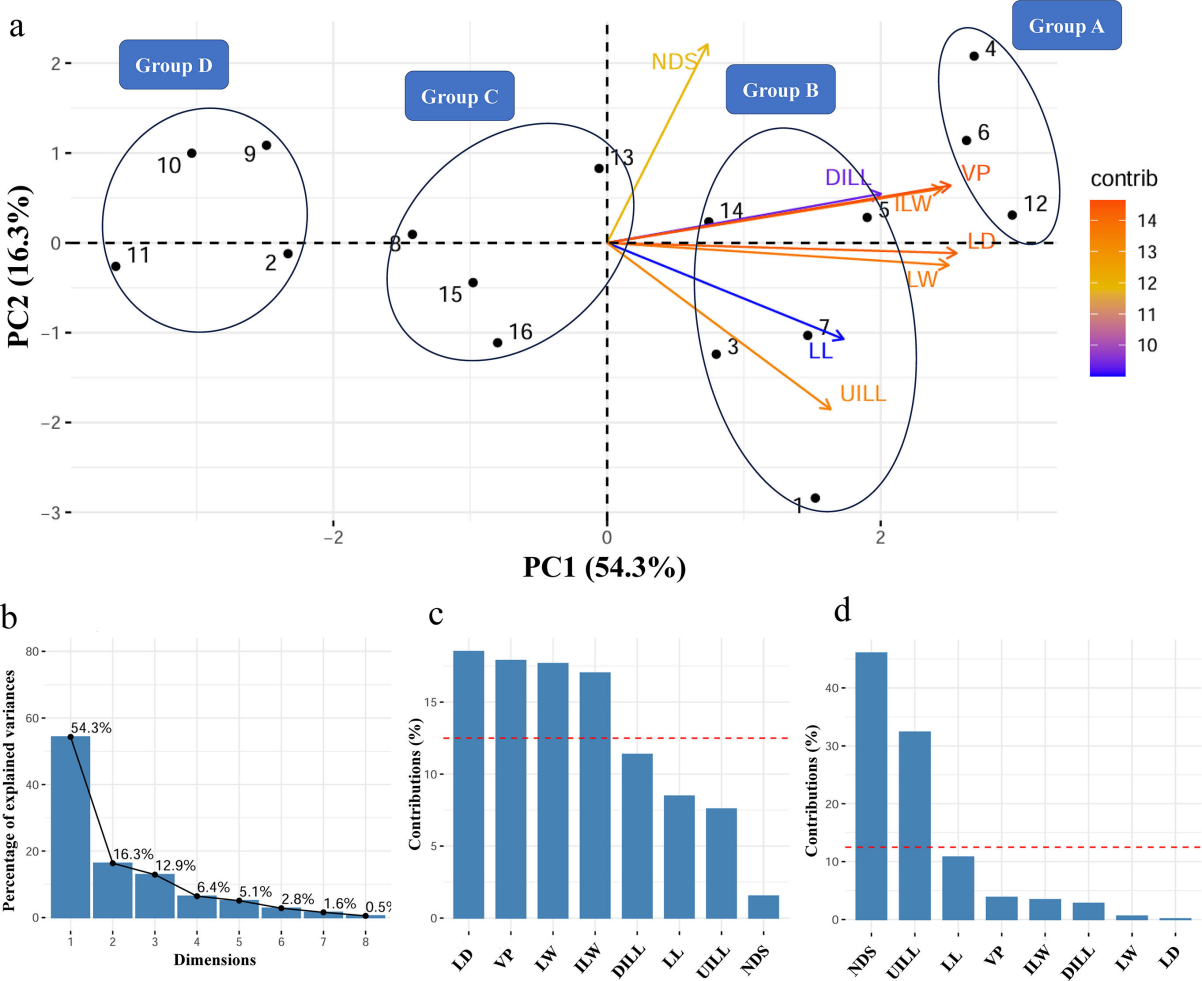
Variability in disease symptom severity among eight pomegranate cultivars inoculated with *Stilbocrea gracilipes* isolates (PM7-68 and EU1-031), as determined by principal component analysis (PCA). (**a**) PCA biplot illustrating the first (PC1) and second (PC2) principal components. These components are derived from eight pathogenicity characteristics assessed two months post-inoculation: lesion length (LL), lesion width (LW), upward internal lesion length (UILL), downward internal lesion length (DILL), internal lesion width (ILW), lesion depth (LD), vascular progression (VP), and number of days exhibiting symptoms over two months (NDS, equivalent to 60 days minus the incubation period). Cultivar responses to the two *S*. *gracilipes* isolates were clustered into four distinct groups based on disease symptom severity, ranging from level 1 (least severe) to level 4 (most severe): Group A (level 4 severity): 4, ‘Malas-e-Danesiyah-e-Esfahani’ inoculated with PM7-68; 6, ‘Shirin-e-Shahvar’ inoculated with PM7-68; and 12, ‘Malas-e-Danesiyah-e-Esfahani’ inoculated with EU1-031; group B (level 3 severity): 1, ‘Rabab-e-Neyriz’ inoculated with PM7-68; 3, ‘Bihaste Ravar’ inoculated with PM7-68; 5, ‘Malas-e-Saveh’ inoculated with PM7-68; 7, ‘Wonderful’ inoculated with PM7-68; and 14, ‘Shirin-e-Shahvar’ inoculated with EU1-031; group C (level 2 severity): 8, ‘Atabaki’ inoculated with PM7-68; 13, ‘Malas-e-Saveh’ inoculated with EU1-031; 15, ‘Wonderful’ inoculated with EU1-031; and 16, ‘Atabaki’ inoculated with EU1-031; and Group D (level 1 severity): 2, ‘Kadro’ inoculated with PM7-68; 9, ‘Rabab-e-Neyriz’ inoculated with EU1-031; 10, ‘Kadro’ inoculated with EU1-031; and 11, ‘Bihaste Ravar’ inoculated with EU1-031. (**b**) Percentage of variance explained by each of the eight principal components. (**c and d**) Bar graphs depicting the percentage contribution of each variable (pathogenicity traits: LL, LW, UILL, DILL, ILW, LD, VP, and NDS) to the first (**c**) and second (**d**) principal components.

**Fig 12:**
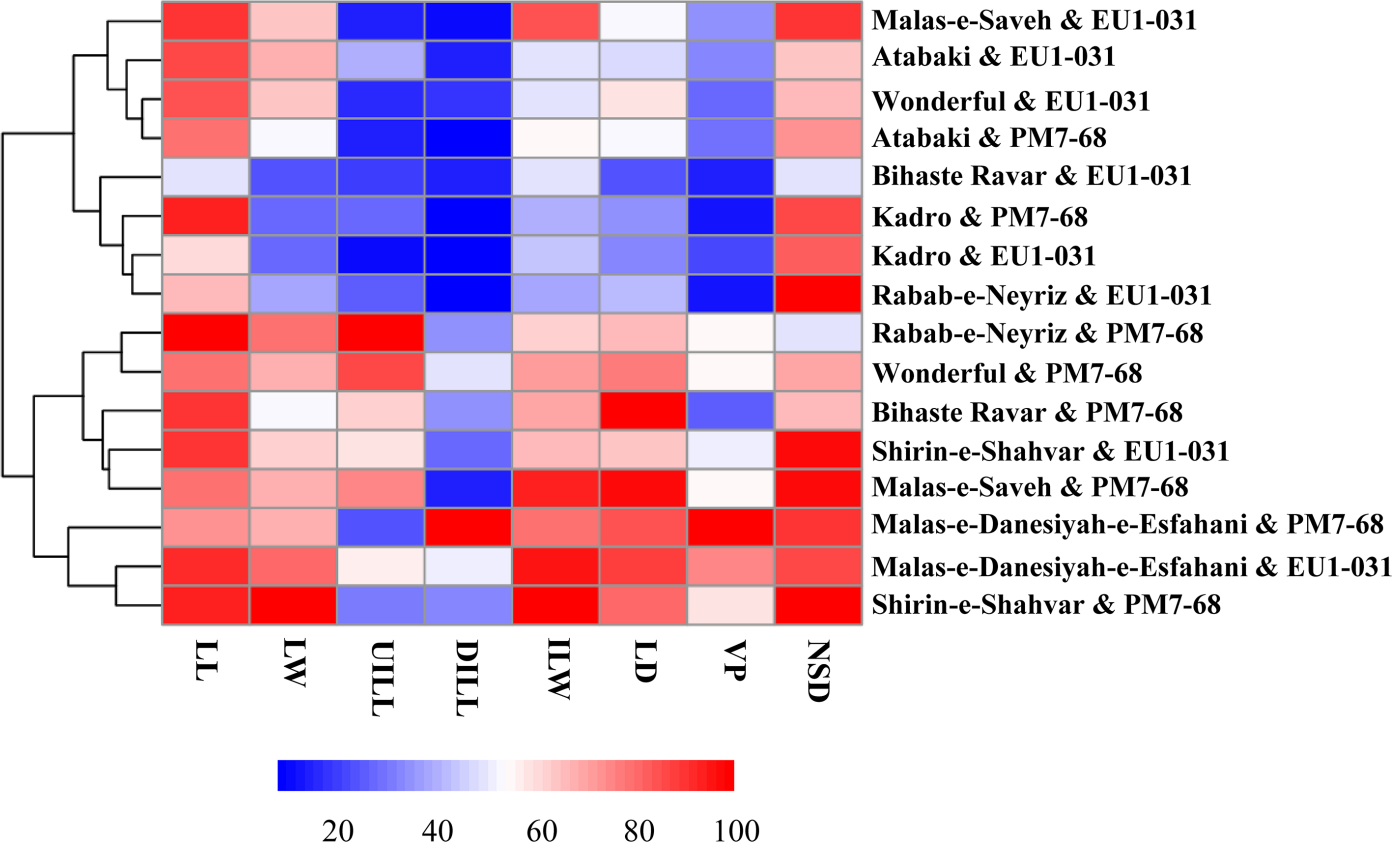
Heatmap depicting the relative severity of eight pathogenicity traits assessed on inoculated saplings of eight pomegranate cultivars inoculated with *Stilbocrea gracilipes* isolates (PM7-68 and EU1-031). Pathogenicity traits included wood lesion length (LL), wood lesion width (LW), upward (UILL), downward internal wood lesion length (DILL), internal lesion width (ILW), lesion depth (LD), vascular progression (VP), and the number of days exhibiting symptoms over two months (NDS). The NDS value was calculated as 60 days minus the incubation period. Severity scores for each trait were normalized using the following formula: (Observed value for trait / Maximum observed value for trait) × 100.

Principal component analysis (PCA) of eight pathogenicity traits revealed distinct clustering patterns in the disease severity of cultivars inoculated with *S. gracilipes* isolates (Fig. 11). Based on the severity of disease symptoms, cultivar responses to the two *S*. *gracilipes* isolates clustered into four distinct groups, ranging from level 1 (least severe) to level 4 (most severe). Group A (level 4) comprised ‘Malas-e-Danesiyah-e-Esfahani’ inoculated with PM7-68, ‘Shirin-e-Shahvar’ inoculated with PM7-68, and ‘Malas-e-Danesiyah-e-Esfahani’ inoculated with EU1-031. Group B (level 3) included ‘Rabab-e-Neyriz’, ‘Bihaste Ravar’, ‘Malas-e-Saveh’, and ‘Wonderful’ inoculated with PM7-68, as well as ‘Shirin-e-Shahvar’ inoculated with EU1-031. Group C (level 2) consisted of ‘Atabaki’ inoculated with PM7-68 and ‘Malas-e-Saveh’, ‘Wonderful’, and ‘Atabaki’ inoculated with EU1-031. Group D (level 1) contained ‘Kadro’ inoculated with PM7-68, and ‘Rabab-e-Neyriz’, ‘Kadro’, and ‘Bihaste Ravar’ inoculated with EU1-031. A heatmap of disease severity, based on the eight pathogenicity traits, clustered all cultivar-isolate interactions into four distinct clusters, corroborating the PCA clustering pattern (Fig. 12).

## DISCUSSION

The increasing incidence and prevalence of syndromes characterized by canker development, dieback, and overall decline in pomegranate trees within the Fars Province, a key pomegranate production area in Iran, have prompted us to investigate the fungal agents associated with these manifestations. This study provides the first detailed global report of *S. gracilipes* associated with stem cankers, twig dieback, and internal wood discoloration in pomegranate trees, as well as in landscape eucalyptus (*Eucalyptus camaldulensis*) trees. Through morphological, physiological, and multi-locus phylogenetic analyses, utilizing ITS, *rpb2*, and *tef1-α* loci, we confirmed that the isolates obtained from symptomatic trees constitute a distinct and strongly supported monophyletic lineage within the genus *Stilbocrea* (*Bionectriaceae*), closely related to *S. gracilipes* isolates. *Stilbocrea gracilipes* is commonly found in pantropical, warm temperate, and temperate zones, colonizing diverse substrates such as the bark and wood of various plant species exhibiting dieback symptoms (37–42, 47). Although none of the previous studies report the cardinal temperatures of this species, our findings indicate that *S. gracilipes* exhibits optimal mycelial growth between 25°C (for pomegranate isolates) and 30°C (for eucalyptus isolates), with the ability to grow at temperatures up to 35°C. Therefore, it seems that this fungus is thermotolerant, which may contribute to its widespread distribution across pantropical and temperate regions.

The comprehensive pathogenicity test, encompassing all recovered *S. gracilipes* isolates, confirmed the ability of these isolates to cause characteristic dark brown wood lesions and internal wood discoloration in their respective host species. Initial pathogenicity assessments, conducted via detached shoot inoculation, demonstrated that all isolates were pathogenic, albeit with significant variation in aggressiveness. Specifically, isolates PM7-68 (from pomegranate) and EU1-031 (from eucalyptus) exhibited the highest levels of aggressiveness, producing statistically significant and more extensive lesions on detached shoots of their respective hosts compared to other isolates. The detached shoot assay proved to be an efficient method for rapidly evaluating isolate aggressiveness based on quantitative traits, including upward and downward wood lesion progression and wood lesion width. This inoculation approach aligns with previously validated methodologies and is a widely used technique in similar studies (e.g., 34, 48–52). The detached shoot inoculation method, while effective for initial screening, is conducted on disinfected plant tissue under controlled temperature and humidity, which may not fully mimic the complexities of natural infection processes (53). Therefore, to complement these findings and to better reflect natural conditions, representative aggressive isolates from both pomegranate and eucalyptus were selected for subsequent pathogenicity tests on pomegranate and eucalyptus saplings.

Building on the initial pathogenicity screening results, the cross-pathogenicity assessments of *S. gracilipes* isolates on pomegranate and eucalyptus saplings demonstrated that isolates from original hosts were pathogenic on both plant species, inducing characteristic internal wood discoloration. However, isolates generally exhibited greater pathogenicity on their original host species. This capacity to cause disease on taxonomically distant hosts indicates a lack of strict host specificity, raising concerns about the pathogen’s ecological plasticity and potential for dissemination (54). Factorial ANOVA conducted on the cross-pathogenicity data set revealed significant differences in aggressiveness between *S. gracilipes* isolates originating from pomegranate and eucalyptus, as well as differential susceptibility of the two host species to these isolates. The pomegranate isolate PM7-68 exhibited a higher level of aggressiveness compared to the eucalyptus isolate EU1-031 based on the evaluation of pathogenicity traits. Furthermore, inoculated pomegranate saplings demonstrated greater susceptibility to this pathogen than inoculated eucalyptus saplings, based on eight pathogenicity traits, highlighting the potential threat of *S. gracilipes* to economically important pomegranate orchards in Fars Province. These cross-pathogenicity results are consistent with previous findings regarding the emerging canker pathogen, *Stilbocrea banihashemiana* Bolboli, Tavakolian & Mostowf., which affects fruit and landscape ornamental trees, suggesting that such host generalism may be more widespread among tropical or subtropical canker pathogens than previously recognized (35, 55).

In assessing the susceptibility of pomegranate cultivars to the emerging pathogen *S. gracilipes*, our study employed comprehensive statistical analysis, including PCA and heatmap visualization, to discern variations in disease response. PCA revealed significant differences in susceptibility among the eight tested pomegranate cultivars to *S. gracilipes* isolates PM7-68 (from pomegranate) and EU1-031 (from eucalyptus). This conclusion was based on a thorough evaluation of multiple pathogenicity traits, including external lesion dimensions, internal discoloration, VP, and IP. The selection of pomegranate cultivars for this study was guided by their economic importance. Specifically, ‘Malas-e-Saveh’ and ‘Rabab-e-Neyriz’ were included due to their prominence in Iran, where they are highly valued for their superior flavor, fruit size, and productivity (56). Additionally, the ‘Wonderful’ cultivar was incorporated, as it represents the most globally cultivated variety, particularly in the United States, due to its substantial yields and consumer appeal (57, 58). PCA and heatmap visualization indicated that the severity of disease symptoms in cultivar-isolate combinations clustered into four distinct groups, ranging from level 1 (least severe) to level 4 (most severe). ‘Malas-e-Danesiyah-e-Esfahani’ consistently exhibited higher levels of disease severity (level 4) in response to both *S. gracilipes* isolates, thereby identifying it as a highly susceptible cultivar. Conversely, ‘Kadro’ displayed reduced lesion development and slower disease progression (level 1), suggesting relative resistance to both *S. gracilipes* isolates. The majority of isolate-cultivar combinations clustered in levels 2 and 3 of disease severity, exhibiting moderate symptoms. This stratification underscores the complex nature of *S. gracilipes* isolate and pomegranate cultivar interactions. The observed variation in cultivar susceptibility emphasizes the importance of informed cultivar selection in effective disease management programs. Furthermore, PCA results indicated that LD and VP in PC1 (54.3%), along with the number of days with symptoms (NSD) and upward internal lesion length (UILL) in PC2 (16.3%), were the most critical traits in cultivar susceptibility assessments. These traits were the key contributors to the variance explained by PC1 and PC2. The PCA biplot revealed a strong relationship between the pathogenicity traits DILL, VP, and ILW, as well as between LD and LW. Conversely, the pathogenicity traits UILL and NDS demonstrated a weaker relationship within the cultivar susceptibility assessment data set.

To the best of our knowledge, this study represents the first report of *S. gracilipes* associated with canker and dieback symptoms on pomegranate and eucalyptus, as well as the first confirmation of its pathogenicity as a canker-causing fungus on woody hosts both in Iran and worldwide. This identification enhances our understanding of emerging fungal pathogens in tropical and subtropical agroecosystems and underscores the critical need for ongoing surveillance and early detection strategies. The ability of *S. gracilipes* to infect both fruit and ornamental trees, coupled with its demonstrated cross-host infectivity, presents a potential risk to both mixed cropping systems and natural ecosystems. This threat is particularly pronounced under stress conditions, such as drought or mechanical injury, which are known to intensify canker disease severity (58). The isolation of this emerging pathogen from ornamental eucalyptus trees in urban landscapes, in close proximity to pomegranate orchards in this study, as well as the isolation of this pathogen from dieback symptoms in black locust, chico, and saxaul trees in previous investigations, suggests the potential relevance of the Bridgehead effect theory (40–42). This theory posits that pathogens can utilize a new environment or host plant as a primary base from which to invade and incite epidemics when conditions are favorable (59). Urban trees may serve as suitable establishment sites for invasive pathogens that have been inadvertently introduced into a new area. The Bridgehead effect likely plays a significant role in facilitating long-term damage and triggering costly management efforts associated with plant pathogens. Furthermore, the observed cultivar-dependent susceptibility to this emerging pathogen emphasizes the importance of integrating resistant genotypes into orchard design and breeding programs. Future research should prioritize defining the potential host range of this emergent pathogen, elucidating the molecular mechanisms underlying cultivar defense responses, assessing the feasibility of pathogen detection in other trees within infected regions, and developing climate-adaptive disease management strategies to minimize its potential impact on the region’s horticultural and forestry systems.

## MATERIALS AND METHODS

### Sampling and fungal isolation

During the period of 2024–2025, pomegranate and eucalyptus trees displaying trunk and stem canker symptoms were surveyed across various locations in Shiraz and Arsanjan counties, Fars Province, in southern Iran. Sample collection encompassed commercial pomegranate orchards and adjacent landscapes, and naturally growing eucalyptus trees. Branches and trunks exhibiting characteristic symptoms, including wood discoloration and bark cracking, were collected for fungal isolation. Wood tissue samples (25 mm²) were aseptically excised from the margins of discolored and necrotic wood tissues. These fragments were initially rinsed under running tap water for 5 min to remove any adhering debris. Surface disinfection was performed by sequential immersion in 70% ethanol for 1 min, followed by 0.5% sodium hypochlorite (10% commercial bleach) for 1 min. Samples were then rinsed twice with sterile distilled water for 1 min to remove residual disinfectants. Subsequently, the samples were air-dried on sterile paper towels within a laminar flow hood for 15 min. The sterilized wood fragments were then transferred to PDA (prepared from 300 g/L boiled potato extract, 20 g/L dextrose, 15 g/L agar, and distilled water), which was amended with 1 mg/L tetracycline to inhibit bacterial growth. Plates were incubated at 25 ± 0.5°C in the dark for 7 days. To obtain pure cultures, emerging fungal colonies were subcultured onto water agar (WA; 20 g/L agar and distilled water). Following a 3-day IP at 25 ± 0.5°C, single hyphal tips were isolated from WA and transferred to PDA. The resulting pure cultures were then grown on sterile filter paper and subsequently preserved at −20°C in the culture collection of the Plant Protection Department, Shiraz University, for further analysis (60).

### Morphological investigations and the effect of temperature on mycelial growth

For morphological characterization of *Bionectriaceae*-like isolates, mycelial plugs from pure cultures were transferred to PDA and malt extract agar (MEA; 20 g/L malt extract, 15 g/L agar, and distilled water). Cultures were then incubated at 25 ± 0.5°C under a 12-hour light/dark cycle for one week. Key colony traits, including morphology and pigmentation on both PDA and MEA, were examined alongside microscopic structures, such as conidiophore arrangement, phialide morphology and size, and conidial shapes and dimensions (37, 38). Microscopic measurements were conducted on representative isolates (APM2-01, EU1-031, and PM7-68) according to their host species and geographic origin. Fungal structures were mounted in lactic acid, and measurements were taken of at least 30 instances of each specified microscopic structure per isolate. Temperature-growth relationships and average growth rates were assessed for selected isolates on 80-mm Petri dishes containing 25 mL of PDA and MEA at incubation temperatures of 5, 10, 15, 20, 25, 30, 35, and 40 ± 0.5°C. Colony diameters were measured after 10 days, using four replicates per isolate at each temperature.

### Molecular studies

For molecular characterization, representative isolates from both hosts (pomegranate: APM2-01, and PM7-68; eucalyptus: EU1-031) were cultured in potato broth (PB; 300 g/L boiled potato extract in distilled water) and incubated for 15 days. Mycelia were then harvested, lyophilized, and genomic DNA was extracted using the DNG-PLUS extraction kit (CinnaGen, Tehran, Iran), following the manufacturer’s instructions. A NanoDrop MD-1000 spectrophotometer (NanoDrop Technologies, Wilmington, DE, USA) was utilized to evaluate the concentration and purity of the extracted DNA. Polymerase chain reaction (PCR) amplification was performed targeting the internal transcribed spacer one and two regions, including the 5.8S gene of rDNA (ITS) using ITS1/ITS4 primers (61), the translation elongation factor 1-α (*tef1-α*) gene using EF1-728F/EF1-2218R primers (62, 63), and the second largest subunit of RNA polymerase II (*rpb2*) gene using fRPB2-5F/fRPB2-7cR primers (64). Each 25 µL PCR reaction contained 1 µL of genomic DNA (~100 ng), 1 µL of each forward and reverse primer (10 pM), 12.5 µL of Taq DNA Polymerase 2× Master Mix RED (Amplicon, Odense, Denmark), and 9.5 µL of PCR-grade water. Amplifications were conducted using a Peltier Thermal Cycler (Bio-Techne, Minneapolis, MN, USA) under thermocycling conditions optimized for each primer set (Table S1). The PCR results were validated through electrophoresis on a 1% agarose gel containing 0.05% ethidium bromide. PCR products were sequenced at the Cardiogenetic Research Center (Tehran, Iran) using the same primers as those used for amplification, employing a dye terminator cycle sequencing protocol. The sequence data generated in this study have been deposited in GenBank (http://www.ncbi.nlm.nih.gov/genbank).

### Phylogenetic analyses

Newly generated sequences were edited and aligned using BioEdit v. 7.0.9.1 (65). To identify closely related sequences, a BLASTn search was performed against the NCBI GenBank nucleotide database to find sequences with high similarity to representative isolates (APM2-01, EU1-031, and PM7-68) from infected pomegranate and eucalyptus trees, facilitating preliminary phylogenetic classification. The phylogenetic analyses included 27 ingroup taxa for ITS and 23 ingroup taxa for *rpb2*, *tef1-α*, and the combined alignment (Table S2). *Thyronectria rhodochlora* (Mont.) Seeler (CBS 136006) was designated as the outgroup for all phylogenetic analyses. Multiple sequence alignments were performed using MAFFT version 7 (66), with manual adjustments made where necessary to optimize the alignment. Prior to the combined analyses, a partition homogeneity test was conducted on the concatenated nuclear gene alignment using PAUP v. 4.0a136 (66, 67) with 100 replicates and a heuristic search. BI analyses were conducted on individual and concatenated ITS, *rpb2*, and *tef1-α* loci using MrBayes 3.1 (68) via the TrEase online platform (69). BI analyses were run for 10 million generations employing a GTR Gamma + I substitution model, with the initial 30% of generations discarded as burn-in. ML analyses were also performed in TrEase using RAxML and the same GTR+1 + G model to assess phylogenetic relationships further. The robustness of the ML trees was evaluated using 1,000 bootstrap replicates. Phylogenetic trees were visualized and refined using MEGA 11 software (70).

### Pathogenicity and aggressiveness assessments of isolates on original hosts

To evaluate the pathogenicity and aggressiveness of *S. gracilipes* isolates (15 isolates from pomegranate and eight isolates from eucalyptus trees) on their respective original hosts, healthy and asymptomatic detached shoots of pomegranate (a commercially important cultivar of ‘Rabab-e-Neyriz’) and eucalyptus (*E. camaldulensis*) trees were collected. Young shoots (20–30 cm in length, 5–7 mm in diameter) were excised and maintained at 4°C during transport to the laboratory. Collected shoots were surface-disinfected by washing with tap water, followed by treatment with 99% ethanol and flaming (for 1 s). A sterile 5 mm cork borer was used to create uniform wounds, into which 5 mm plugs from 10-day-old cultures on PDA were inserted. Inoculation sites were sealed with Parafilm (Bemis Company, USA) to minimize desiccation and prevent contamination, while plugs of non-colonized PDA served as negative controls (71). Each isolate was inoculated onto three detached shoots originating from their respective host, with two inoculation sites per shoot (15 cm apart). The basal ends of the shoots were then submerged in 300 mL of sterile distilled water within Erlenmeyer flasks, and the upper ends, along with any incidental wounds, were sealed with paraffin to ensure hydration. Inoculated shoots were incubated in a controlled environment chamber at 25 ± 1°C under a 16-hour light/8-hour dark cycle, arranged in a completely randomized design (CRD). Fifteen days post-inoculation, the bark tissue was dissected to assess ULP and DLP, as well as LW, in each replicate (*n* = 6). These measurements were obtained using a digital caliper (INSIZE, 0.01 mm accuracy). To fulfill Koch’s postulates, small fragments of necrotic tissue from lesion margins were transferred to PDA amended with tetracycline (100 mg/L). Resulting fungal colonies were then examined for morphological characteristics to confirm pathogen identity.

### Cross-pathogenicity tests on one-year-old saplings of original hosts

To investigate the cross-pathogenicity of *S. gracilipes* isolates and assess host species susceptibility, a controlled experiment was conducted. Two aggressive isolates, previously identified via detached shoot inoculations (PM7-68 from pomegranate and EU1-031 from eucalyptus), were selected for this study. One-year-old saplings of *E. camaldulensis* and *P. granatum* cv. ‘Rabab-e-Neyriz’, representing the original hosts of the respective isolates, were reciprocally inoculated. Saplings were inoculated by inserting a 5 × 5 × 2 mm mycelial plug, excised from the actively growing margin of a 9-day-old fungal culture on PDA, into a 5 × 5 mm incision created on the sapling’s main stems at the midpoint, between two nodes, following bark removal using a sterile scalpel. Inoculation sites were then wrapped with Parafilm to prevent desiccation and contamination. Non-colonized PDA plugs were used as negative controls. The experiment was arranged in a CRD under greenhouse conditions, with three saplings per host species and two inoculation points per sapling (*n* = 6). Symptom development was monitored for two months following inoculation. At the end of this period, eight pathogenicity traits were evaluated in inoculated pomegranate and eucalyptus saplings for both isolates. These traits included LL, LW on the bark, and UILL and DILL, as well as ILW after bark removal, LD after cross-sectioning inoculated stems, VP based on wood discoloration along a longitudinal section, and IP (defined as the number of days between inoculation and the appearance of disease symptoms). All lesion measurements were taken using a digital caliper (INSIZE, 0.01 mm accuracy). To confirm Koch’s postulates, 18 woody pieces (25 mm²) from the margin of symptomatic tissues in each treatment (three pieces from each replicate) were excised and cultured on PDA supplemented with tetracycline. The recovered isolates were subsequently examined for morphological confirmation.

### Assessing the susceptibility of different pomegranate cultivars to *S. gracilipes*

To evaluate the susceptibility of various pomegranate cultivars to *S. gracilipes*, eight commercially significant one-year-old pomegranate cultivars—‘Rabab-e-Neyriz’, ‘Kadro’, ‘Bihaste Ravar’, ‘Malas-e-Danesiyah-e-Esfahani’, ‘Malas-e-Saveh’, ‘Shirin-e-Shahvar’, ‘Wonderful’, and ‘Atabaki’ —were selected (72). These cultivars were inoculated with two *S*. *gracilipes* isolates: one derived from pomegranate (PM7-68) and another from eucalyptus (EU1-031), both of which had been previously determined to be aggressive in detached shoot assays. Each cultivar was separately inoculated with both the pomegranate-obtained and eucalyptus-obtained isolates, resulting in a total of 16 treatments, each with three biological replicates (one-year-old saplings). The inoculation procedure, consistent with the method described in the preceding section, involved stem wounding followed by the application of mycelial plugs. Three saplings of each cultivar were inoculated with sterile PDA plugs as negative controls, and symptoms induced by both pathogen isolates were compared against these controls. The experiment was conducted under greenhouse conditions, arranged in a CRD. Plants were routinely irrigated to maintain optimal soil moisture levels, and symptom development was monitored daily for two months. At the end of this period, disease symptoms were assessed, and eight pathogenicity traits (LL, LW, UILP, DILP, ILW, LD, VP, and IP) were documented.

### Statistical analyses

The distribution of pathogenicity traits evaluated in each experiment (isolate aggressiveness, cross-pathogenicity, and cultivar susceptibility) was tested for normality using the Shapiro–Wilk test (73) in IBM SPSS Statistics v.25. When necessary, data were transformed to meet the assumptions of parametric analyses. For aggressiveness assessments, the effect of isolates on pathogenicity traits was analyzed using one-way ANOVA within the general linear model framework in SAS v.9.4 (SAS Institute Inc., Cary, NC, USA). For cross-pathogenicity tests, factorial ANOVA was employed to examine the main effects of isolate and host species, as well as their interaction. Similarly, factorial ANOVA was used in cultivar susceptibility assessments to evaluate the main effects of isolate and cultivar, along with their interaction. Mean separation was performed using Tukey’s Honestly Significant Difference (HSD) test at a significance threshold of *P* ≤ 0.05.

PCA was conducted in R v.3.4.0 (http://www.r-project.org) to visualize clustering patterns and variance in disease severity across the 16 treatments, based on the eight recorded pathogenicity traits in the cultivar susceptibility experiment. To ensure positive correlations among pathogenicity traits and cultivar classification in PCA, the number of days exhibiting symptoms within two months (NDS; 60-day IP) was used in place of the IP itself. Heatmaps were also generated in R v.3.4.0 to depict the relative severity of pathogenicity traits across cultivars. Prior to heatmap construction, trait values were normalized by dividing each observation by the maximum recorded value for that trait and multiplying by 100, thereby expressing severity as percentages.

## Data Availability

The data that support the findings of this study are openly available from the corresponding author upon reasonable request. The DNA sequencing data have been deposited in the National Center for Biotechnology Information (https://www.ncbi.nlm.nih.gov/) under accession numbers PV929588, PV935455, PV930043, PV929590, PV935456, PV930045, PV929589, PV935457, and PV930044.

## SUPPLEMENTAL MATERIAL

Supplemental material spectrum.02839-25-s0001.DOCX Tables S1 to S6 and Fig. S1 to S3.

Graphical abstract spectrum.02839-25-s0002.tif Visual depiction of the study.

ASM does not own the copyrights to Supplemental Material that may be linked to, or accessed through, an article. The authors have granted ASM a non-exclusive, world-wide license to publish the Supplemental Material files. Please contact the corresponding author directly for reuse.
