# Supplementary material for: Emergence of Stilbocrea gracilipes associated with canker and dieback in pomegranate and eucalyptus trees and host-specific responses
Source: Microbiol Spectr. 2026 Feb 3;14(3):e02839-25. doi: 10.1128/spectrum.02839-25 (PMC12955467; doi:10.1128/spectrum.02839-25)
Supplement: Supplemental material — Tables S1 to S6 and Fig. S1 to S3. [file spectrum.02839-25-s0001.docx]

**Supplementary Table 1** PCR conditions used in this study.

| **Gene 1** | **Number of Cycles** | **Initial Denaturation** | **Denaturation** | **Annealing** | **Extension** | **Final Extension** |
| --- | --- | --- | --- | --- | --- | --- |
| ITS | 30 | 95 (120) | 95 (45) | 64 (45) | 72 (45) | 72 (600) |
| *tef1* | 35 | 95 (120) | 95 (120) | 65 (60) | 72 (50) | 72 (600) |
| *rpb2* | 35 | 95 (120) | 95 (120) | 66.5 (60) | 72 (45) | 72 (600) |

ITS = internal transcribed spacers 1 and 2 and 5.8 S gene of rDNA; *tef1* = translation elongation factor 1-α; *rpb2 =* the second largest subunit of RNA polymerase II.

**Supplementary Table 2** GenBank accession numbers for the isolates included in the phylogenetic analysis in this study.

| **Species** | **Isolate** | **Isolation source** | **Location** |  | **GenBank accession number^a^** | | |
| --- | --- | --- | --- | --- | --- | --- | --- |
|  |  |  |  |  | **ITS** | ***tef1*** | ***rpb2*** |
| *Alloacremonium humicola* | CBS 613.82 | agricultural soil | Netherlands |  | OQ429496 | OQ470786 | OQ453888 |
| *Bulbithecium hyalosporum* | CBS 318.91 | Dung of horse | Peru |  | OQ429508 | OQ470797 | OQ453897 |
| *Bulbithecium pinkertoniae* | CBS 157.70 | Soil from tropical greenhouse | Netherlands |  | OQ429509 | OQ470799 | OQ453898 |
| *Geosmithia pallida* | CBS 101067 | starch | Netherlands |  | PV272730 | PV273510 | PV273309 |
| *Geosmithia putterillii* | CBS 233.38 | decaying timber | New Zealand |  | KT155839 | PV273509 | PV273308 |
| *Hapsidospora irregularis* | CBS 510.70 | Lawn grass compost heap | Canada |  | MH859824 | OQ470968 | OQ454058 |
| *Heleococcum aurantiacum* | CBS 201.35 | - | - |  | MH855645 | JX158397 | JX158463 |
| *Heleococcum japonense* | CBS 397.67 | wood in seawater | Japan |  | JX158420 | JX158398 | JX158464 |
| *Hydropisphaera peziza* | CBS 138712 | wood and bark of populus | France |  | PV272849 | PV273624 | PV273410 |
| *Ovicillium subglobosum* | CBS 101963 | soil | Hong Kong |  | OQ429759 | OQ471085 | OQ454170 |
| *Ovicillium oosporum* | CBS 110151 | *Theobroma gileri* | South America |  | OQ429758 | OQ471084 | OQ454169 |
| *Ovicillium attenuatum* | CBS 399.86 | Dead mite on *Auricularia* sp. | Cuba |  | KU382191 | PV273481 | OQ454168 |
| *Stilbocrea banihashemiana* | CBS 148864 | *Ficus carica* | Iran |  | OM615399 | OM876865 | OM876872 |
| *Stilbocrea banihashemiana* | Gh093-1 | *Eriobotrya japonica* | Iran |  | OM615379 | OM876867 | OM908433 |
| *Stilbocrea colubrensis* | CBS 580.73 | *dendrocalamus*, dead leaf | India |  | PV272691 | PV273471 | PV273279 |
| *Stilbocrea colubrensis* | CBS 141857 | dead bambou | France |  | PV272690 | - | PV273278 |
| *Stilbocrea gracilipes* | CBS 657.83 | - | New Zealand |  | PV272693 | PV273473 | PV273281 |
| *Stilbocrea gracilipes* | CBS 301.96 | soil along the coast | Papua New Guinea |  | PV272692 | PV273472 | PV273280 |
| *Stilbocrea gracilipes* | MFLUCC:17-2614 | twig | China |  | ON230052 | ON238004 | - |
| *Stilbocrea gracilipes* | APM2-01 | *Punica granatum* | Iran |  | **PV929588** | **PV935455** | **PV930043** |
| *Stilbocrea gracilipes* | PM7-68 | *P. granatum* | Iran |  | **PV929590** | **PV935456** | **PV930045** |
| *Stilbocrea gracilipes* | EU1-031 | *Eucalyptus camaldulensis* | Iran |  | **PV929589** | **PV935457** | **PV930044** |
| *Stilbocrea hydei* | YYH240722105 | on an unidentified stick | China |  | PQ849524 | - | - |
| *Stilbocrea macrostoma* | CBS 114375 | - | New Zealand |  | OQ429873 | OQ471205 | OQ454272 |
| *Stilbocrea walteri* | CBS 144627 | *Quercus ilex* | Portugal |  | MH562717 | MH562714 | MH577042 |
| *Stilbocrea yunnanensis* | CGMCC 3.25074 | dead wood | China |  | PV154035 | - | - |
| *Stilbocrea yunnanensis* | CGMCC 3.25075 | dead wood | China |  | PV154036 | - | - |
| *Thyronectria rhodochlora ^b^* | CBS 136006 |  |  |  | KJ570704 | KJ570771 | KX514394 |

Note: GenBank accession numbers in bold were newly generated in this study.

^a.^ ITS: internal transcribed spacers 1 and 2 and 5.8S gene of rDNA; *tef1*: translation elongation factor 1-α; *rpb2*: the second largest subunit of RNA polymerase II. ^b.^ Outgroup.

**Supplementary Table 3**  One-way ANOVA results for three pathogenicity traits of inoculated one-year-old detached pomegranate shoots with 15 *Stilbocrea gracilipes* isolates from infected pomegranate trees in Fars Province, Iran.

| **S.O.V.** | **df** |  | **Upward lesion progression** | | |  | **Downward lesion progression** | | |  | **Lesion width** | | |
| --- | --- | --- | --- | --- | --- | --- | --- | --- | --- | --- | --- | --- | --- |
|  |  |  | **MS** | **F-value** | ***P*-value** |  | **MS** | **F-value** | ***P*-value** |  | **MS** | **F-value** | ***P*-value** |
| **Isolate** | 14 |  | 0.47 | 125.23 | <0.0001 |  | 27.51 | 213.64 | <0.0001 |  | 4.28 | 86.46 | <0.0001 |
| **Error** | 30 |  | 0.0037 |  |  |  | 0.1287 |  |  |  | 0.0495 |  |  |
| **CV (%)** |  |  | **1.49** | | |  | **3.43** | | |  | **3.95** | | |

CV: Coefficient of variation; df: Degrees of freedom; MS: Mean Square; S.O.V.: Source of variation.

**Supplementary Table 4**  One-way ANOVA results for three pathogenicity traits of inoculated one-year-old detached eucalyptus shoots with 8 *Stilbocrea gracilipes* isolates from infected eucalyptus trees in Fars Province, Iran.

| **S.O.V.** | **df** |  | **Upward lesion progression** | | |  | **Downward lesion progression** | | |  | **Lesion width** | | |
| --- | --- | --- | --- | --- | --- | --- | --- | --- | --- | --- | --- | --- | --- |
|  |  |  | **MS** | **F-value** | ***P*-value** |  | **MS** | **F-value** | ***P*-value** |  | **MS** | **F-value** | ***P*-value** |
| **Isolate** | 7 |  | 450.32 | 1318.17 | <0.0001 |  | 4.03 | 2138.06 | <0.0001 |  | 3.33 | 60.09 | <0.0001 |
| **Error** | 16 |  | 0.3416 |  |  |  | 0.0019 |  |  |  | 0.0554 |  |  |
| **CV (%)** |  |  | **1.02** | | |  | **0.64** | | |  | **3.55** | | |

CV: Coefficient of variation; df: Degrees of freedom; MS: Mean Square; S.O.V.: Source of variation.

**Supplementary Table 5** Factorial ANOVA for eight pathogenicity traits on inoculated one-year-old pomegranate and eucalyptus saplings with two *Stilbocrea gracilipes* isolates

| **S.O.V.** | **df** |  | **Lesion length** | | |  | **Lesion width** | | |  | **Upward internal lesion length** | | |  | **Downward internal lesion length** | | |
| --- | --- | --- | --- | --- | --- | --- | --- | --- | --- | --- | --- | --- | --- | --- | --- | --- | --- |
|  |  |  | **MS** | **F-value** | ***P*-value** |  | **MS** | **F-value** | ***P*-value** |  | **MS** | **F-value** | ***P*-value** |  | **MS** | **F-value** | ***P*-value** |
| **Isolate** | 1 |  | 8.12 | 5305.91 | <0.0001 |  | 4.16 | 1129.89 | <0.0001 |  | 0.83 | 402.54 | <0.0001 |  | 4.72 | 536.12 | <0.0001 |
| **Plant** | 1 |  | 1.30 | 852.41 | <0.0001 |  | 0.47 | 128.63 | <0.0001 |  | 8.69 | 4207.73 | <0.0001 |  | 2.13 | 242.41 | <0.0001 |
| **Isolate × Plant** | 1 |  | 0.57 | 378.46 | <0.0001 |  | 0.13 | 34.82 | <0.0001 |  | 77.99 | 37787.2 | <0.0001 |  | 21.74 | 2470.69 | <0.0001 |
| **Error** | 20 |  | 0.002 |  |  |  | 0.004 |  |  |  | 0.002 |  |  |  | 0.009 |  |  |
| **CV (%)** |  |  |  | **1.00** |  |  |  | **2.29** |  |  |  | **0.84** |  |  |  | **2.23** |  |

Note: CV: Coefficient of variation; df: Degrees of freedom; MS: Mean Square; S.O.V.: Source of variation.

**Supplementary Table 5**  Continue.

| **S.O.V.** | **df** |  | **Internal lesion width** | | |  | **Lesion depth** | | |  | **Vascular progression** | | |  | **Incubation period** | | |
| --- | --- | --- | --- | --- | --- | --- | --- | --- | --- | --- | --- | --- | --- | --- | --- | --- | --- |
|  |  |  | **MS** | **F-value** | ***P*-value** |  | **MS** | **F-value** | ***P*-value** |  | **MS** | **F-value** | ***P*-value** |  | **MS** | **F-value** | ***P*-value** |
| **Isolate** | 1 |  | 0.10 | 41.81 | <0.0001 |  | 0.02 | 0.73 | 0.4017 |  | 36.82 | 23147.6 | <0.0001 |  | 1.08 | 60.2 | <0.0001 |
| **Plant** | 1 |  | 3.75 | 1552.55 | <0.0001 |  | 3.64 | 166.58 | <0.0001 |  | 3.56 | 2240.8 | <0.0001 |  | 1.08 | 59.96 | <0.0001 |
| **Isolate × Plant** | 1 |  | 0.58 | 240.56 | <0.0001 |  | 2.27 | 104 | <0.0001 |  | 45.10 | 28355.2 | <0.0001 |  | 3.91 | 217.57 | <0.0001 |
| **Error** | 20 |  | 0.002 |  |  |  | 0.022 |  |  |  | 0.002 |  |  |  | 0.018 |  |  |
| **CV (%)** |  |  |  | **2.25** |  |  |  | **7.98** |  |  |  | **0.50** |  |  |  | **2.13** |  |

Note: CV: Coefficient of variation; df: Degrees of freedom; MS: Mean Square; S.O.V.: Source of variation.

**Supplementary Table 6** Factorial ANOVA for eight pathogenicity traits on inoculated one-year-old saplings of eight commercially important pomegranate cultivars with two *Stilbocrea gracilipes* isolates

| **S.O.V.** | **df** |  | **Lesion length** | | |  | **Lesion width** | | |  | **Upward internal lesion length** | | |  | **Downward internal lesion length** | | |
| --- | --- | --- | --- | --- | --- | --- | --- | --- | --- | --- | --- | --- | --- | --- | --- | --- | --- |
|  |  |  | **MS** | **F-value** | ***P*-value** |  | **MS** | **F-value** | ***P*-value** |  | **MS** | **F-value** | ***P*-value** |  | **MS** | **F-value** | ***P*-value** |
| **Isolate** | 1 |  | 1.25 | 1040.66 | <0.0001 |  | 1.83 | 859.97 | <0.0001 |  | 39.72 | 20744.5 | <0.0001 |  | 45.86 | 33225 | <0.0001 |
| **Cultivar** | 7 |  | 0.37 | 309.16 | <0.0001 |  | 2.68 | 1257.01 | <0.0001 |  | 7.62 | 3976.83 | <0.0001 |  | 39.42 | 28557.4 | <0.0001 |
| **Isolate × Cultivar** | 7 |  | 1.12 | 930.28 | <0.0001 |  | 0.85 | 396.8 | <0.0001 |  | 24.26 | 12666.2 | <0.0001 |  | 6.72 | 4870.61 | <0.0001 |
| **Error** | 80 |  | 0.001 |  |  |  | 0.002 |  |  |  | 0.002 |  |  |  | 0.001 |  |  |
| **CV (%)** |  |  |  | **0.84** |  |  |  | **1.65** |  |  |  | **0.89** |  |  |  | **0.76** |  |

Note: CV: Coefficient of variation; df: Degrees of freedom; MS: Mean Square; S.O.V.: Source of variation.

**Supplementary Table 6**  Continue.

| **S.O.V.** | **df** |  | **Internal lesion width** | | |  | **Lesion depth** | | |  | **Vascular progression** | | |  | **Incubation period** | | |
| --- | --- | --- | --- | --- | --- | --- | --- | --- | --- | --- | --- | --- | --- | --- | --- | --- | --- |
|  |  |  | **MS** | **F-value** | ***P*-value** |  | **MS** | **F-value** | ***P*-value** |  | **MS** | **F-value** | ***P*-value** |  | **MS** | **F-value** | ***P*-value** |
| **Isolate** | 1 |  | 0.83 | 430.53 | <0.0001 |  | 1.47 | 916.71 | <0.0001 |  | 61.56 | 59795 | <0.0001 |  | 0.003 | 0.17 | 0.6778^ns^ |
| **Cultivar** | 7 |  | 1.00 | 522.28 | <0.0001 |  | 0.40 | 247.01 | <0.0001 |  | 64.31 | 62470 | <0.0001 |  | 1.45 | 83.28 | <0.0001 |
| **Isolate × Cultivar** | 7 |  | 0.20 | 103.41 | <0.0001 |  | 0.26 | 158.6 | <0.0001 |  | 12.98 | 12611.8 | <0.0001 |  | 0.76 | 43.89 | <0.0001 |
| **Error** | 80 |  | 0.002 |  |  |  | 0.002 |  |  |  | 0.001 |  |  |  | 0.017 |  |  |
| **CV (%)** |  |  |  | **2.16** |  |  |  | **3.12** |  |  |  | **0.38** |  |  |  | **2.20** |  |

Note: CV: Coefficient of variation; df: Degrees of freedom; MS: Mean Square; ns: Not significant; S.O.V.: Source of variation.


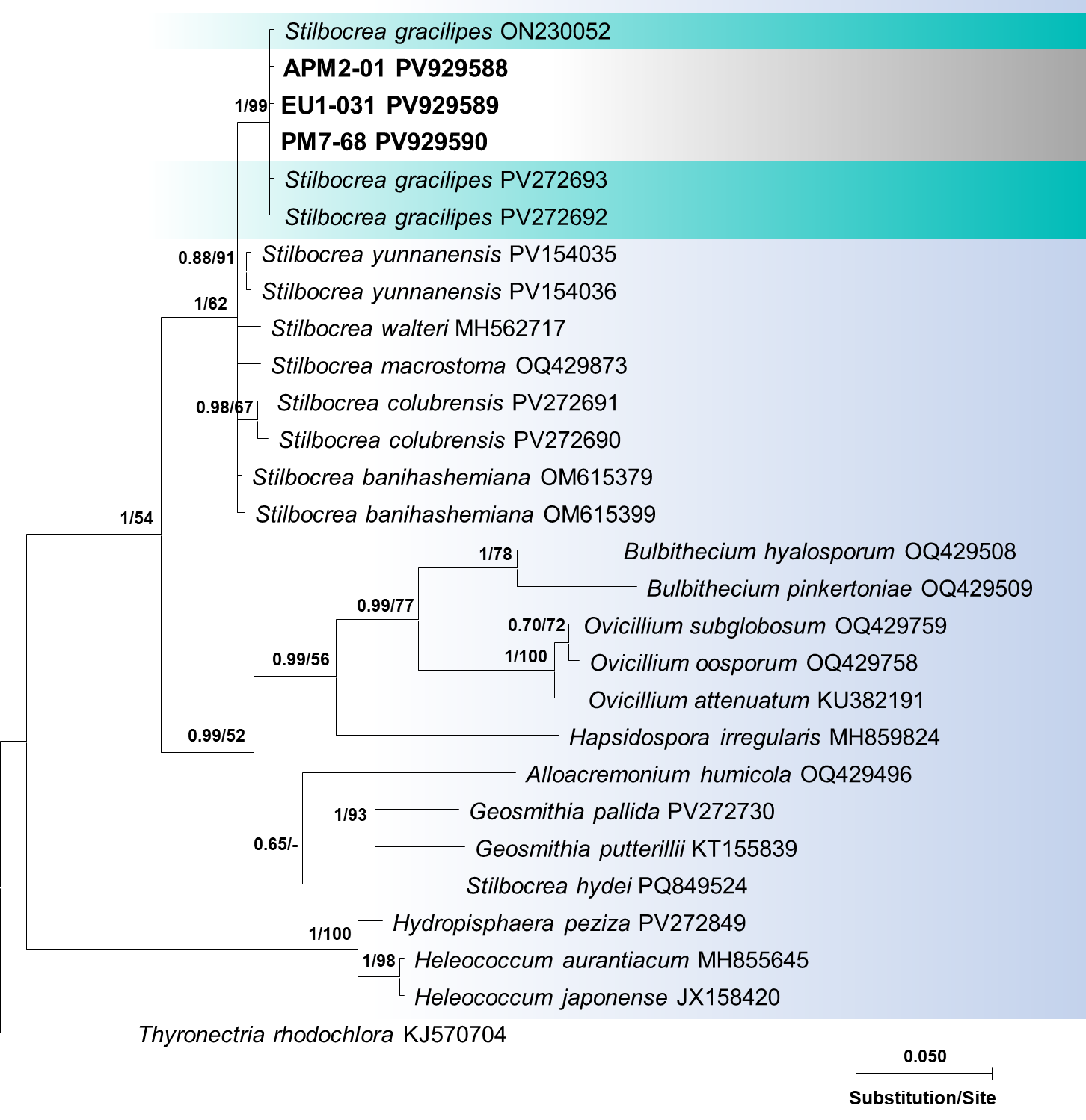


**Supplementary Fig. 1** Phylogenetic relationships of *Stilbocrea gracilipes* isolates obtained from infected pomegranate and eucalyptus trees in southern Iran. The Bayesian tree, based on internal transcribed spacers 1 and 2, the 5.8S gene of rDNA (ITS), illustrates the phylogenetic placement of isolates APM2-01, EU1-031, and PM7-68 within the *Bionectriaceae* family. Bootstrap support values from the maximum likelihood analysis (ML-BS, right) and Bayesian posterior probabilities (BI-PP, left) are shown at the nodes. Branches with ML-BS = 100 and BI-PP = 1 are considered fully supported. The tree was rooted using *Thyronectria rhodochlora* (CBS 136006). Isolates from this study are shown in bold.


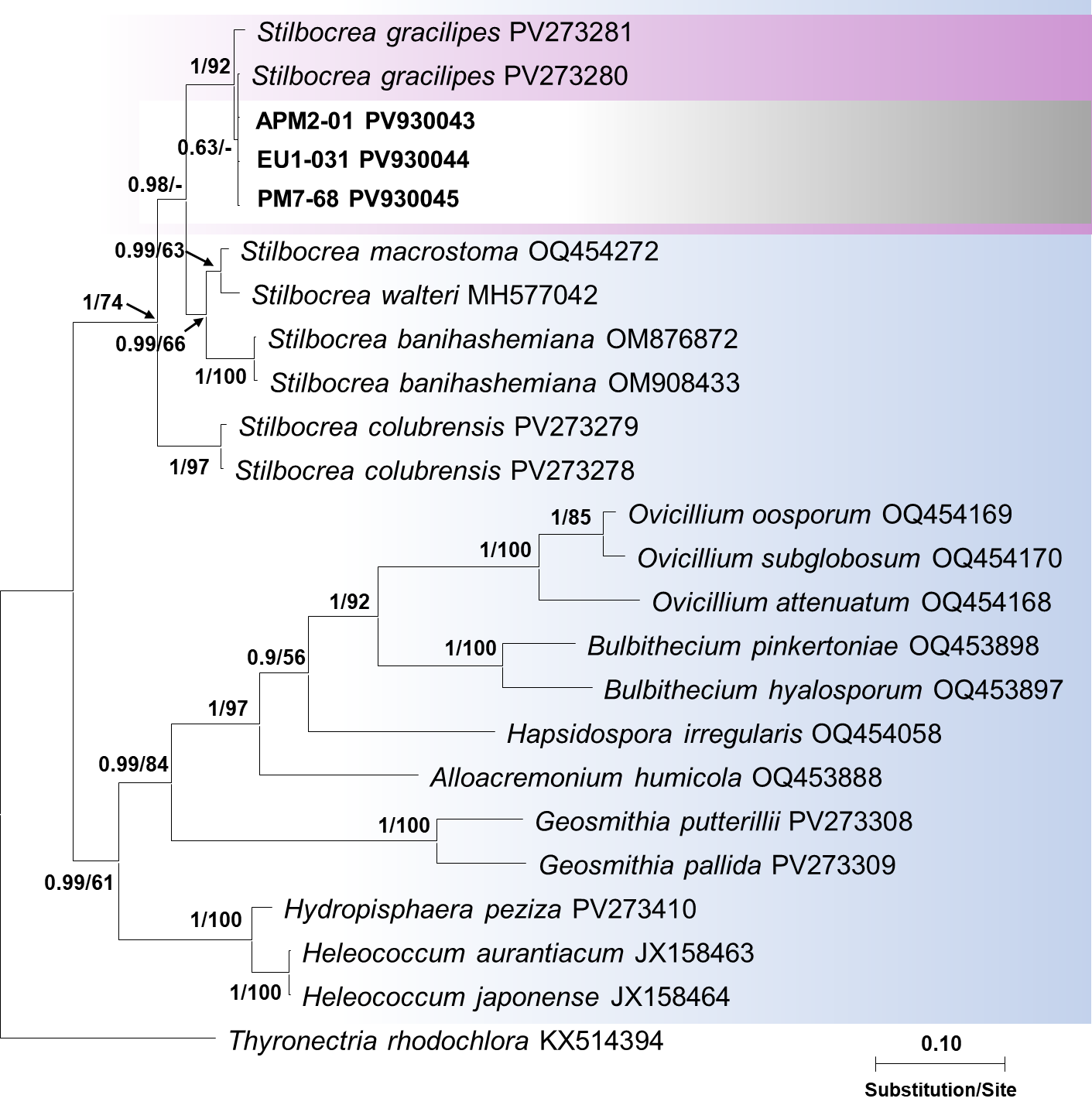


**Supplementary Fig. 2** Phylogenetic relationships of *Stilbocrea gracilipes* isolates obtained from infected pomegranate and eucalyptus trees in southern Iran. The Bayesian tree, based on the second largest subunit of RNA polymerase II (*rpb2*), shows the phylogenetic placement of isolates APM2-01, EU1-031, and PM7-68 within the *Bionectriaceae* family. Bootstrap support values from the maximum likelihood analysis (ML-BS, right) and Bayesian posterior probabilities (BI-PP, left) are shown at the nodes. Branches with ML-BS = 100 and BI-PP = 1 are considered fully supported. The tree was rooted using *Thyronectria rhodochlora* (CBS 136006). Isolates from this study are shown in bold.

**
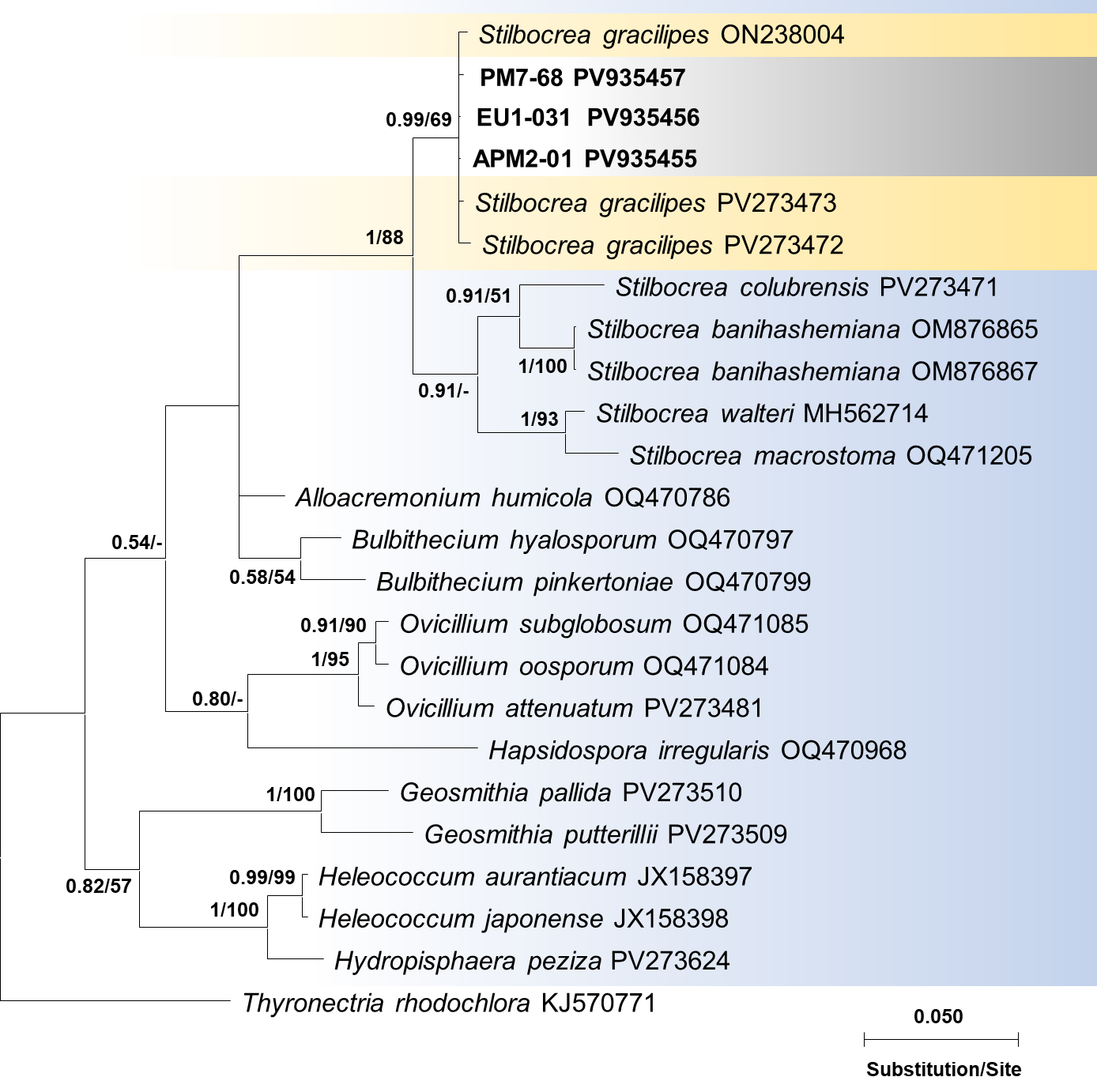
**

**Supplementary Fig. 3** Phylogenetic relationships of *Stilbocrea gracilipes* isolates obtained from infected pomegranate and eucalyptus trees in southern Iran. The Bayesian tree, based on translation elongation factor 1-α (*tef1*), illustrates the phylogenetic placement of isolates APM2-01, EU1-031, and PM7-68 within the *Bionectriaceae* family. Bootstrap support values from the maximum likelihood analysis (ML-BS, right) and Bayesian posterior probabilities (BI-PP, left) are shown at the nodes. Branches with ML-BS = 100 and BI-PP = 1 are considered fully supported. The tree was rooted using *Thyronectria rhodochlora* (CBS 136006). Isolates from this study are shown in bold.
