# Supplementary figures and images for: Emergence of Stilbocrea gracilipes associated with canker and dieback in pomegranate and eucalyptus trees and host-specific responses
Source: Microbiol Spectr. 2026 Feb 3;14(3):e02839-25. doi: 10.1128/spectrum.02839-25 (PMC12955467; doi:10.1128/spectrum.02839-25)

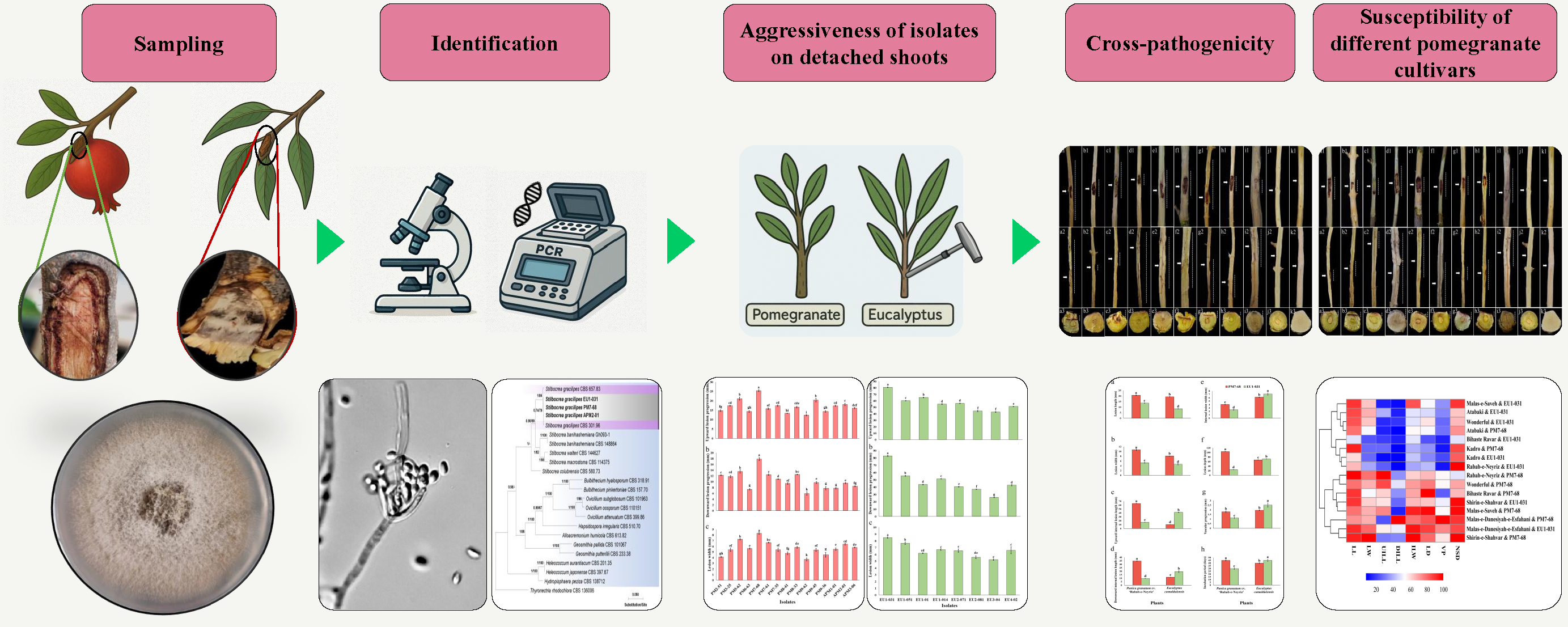

Supplement: Graphical abstract — Visual depiction of the study. [file spectrum.02839-25-s0002.tif]
